# Metabolic Syndrome Predicts Response to Neoadjuvant Chemotherapy in Breast Cancer

**DOI:** 10.3389/fonc.2022.899335

**Published:** 2022-07-01

**Authors:** Ying Lu, Pinxiu Wang, Ning Lan, Fei Kong, Awaguli Abdumijit, Shiyan Tu, Yanting Li, Wenzhen Yuan

**Affiliations:** ^1^ The First School of Clinical Medicine, Lanzhou University, Lanzhou, China; ^2^ The Department of Oncology, The First Hospital of Lanzhou University, Lanzhou, China

**Keywords:** breast cancer, metabolic syndrome, neoadjuvant chemotherapy, efficacy prediction, MP grading, RECIST criteria

## Abstract

**Purpose:**

This research investigated the predictive role of metabolic syndrome (MetS) in breast cancer neoadjuvant chemotherapy (BCNACT) response.

**Methods:**

One hundred fifty primary breast cancer (BC) patients who underwent neoadjuvant chemotherapy (NACT) were included retrospectively. MetS, MetS components [waist circumference (WC), fasting blood glucose (FBG), blood pressure, triglycerides (TG), and high-density lipoprotein cholesterol (HDL-C)], serum lipid, and other MetS-related laboratory indicators within two weeks before BCNACT were evaluated. Univariate, multivariate, and subgroup analyses were performed to determine the predictors of BCNACT pathologic complete response (pCR), clinical response, and pathologic response. The effectiveness of the model was evaluated *via* receiver operating characteristic curve (ROC) and calibration curve. External validation was performed through 135 patients.

**Results:**

Univariate analysis revealed that MetS before BCNACT predicted poor BCNACT response (pCR, P = 0.003; clinical response, P = 0.033; pathologic response, P < 0.001). Multivariate analysis confirmed that MetS before BCNACT predicted lower pCR rate (P = 0.041). Subgroup analysis showed that this relationship was significant in estrogen receptor (ER) (−) (RR = 0.266; 95% CI, 0.074–0.954), human epidermal growth factor 2 (HER2) (−) (RR = 0.833; 95% CI, 0.740–0.939) and TNBC (RR = 0.833; 95% CI, 0.636–0.995). Multivariate analysis of external validation confirmed that pretreatment MetS was associated with a lower pCR rate (P = 0.003), and subgroup analysis also confirmed that this relationship had significant statistical differences in ER (−), HER2 (−), and TNBC subgroups.

**Conclusions:**

MetS before BCNACT predicted a lower pCR rate. Intervention on MetS status, especially in ER (−), HER2 (−), and TNBC subgroups, is expected to improve the response rate of BCNACT further.

## Introduction

Breast cancer (BC) is the most common type of female malignant tumor. Despite the overall incidence of cancer decreasing every year, the incidence of BC continues to increase, and rise in obesity is one of the key factors (1). Neoadjuvant chemotherapy (NACT) is known to shrinkage even eliminate tiny lesions, reduce the chances of distant metastasis, and improve clinical and pathologic response rates. NACT is also an excellent model for evaluating efficacy and looking for potential clinical or biological factors associated with efficacy. With the widespread implementation of breast cancer neoadjuvant chemotherapy (BCNACT), NACT is considered as the standard treatment for locally advanced BC, which has improved the overall survival rate of BC (2). Hence, predicting the response of NACT is helpful to evaluate the prognosis of patients. Moreover, some patients still could not benefit from NACT, subject to the risk of adverse reactions and death risk from chemotherapy, and even show cancer progression while undergoing NACT. Therefore, it is needed to determine predictive indicators which could prejudge whether a BC patient will benefit from NACT (3). These indicators help to achieve individualized treatment, avoid unnecessary chemotherapy-related side effects and death, and indirectly promote the development of new drugs. Furthermore, accurate intervention on predictors could further improve chemotherapy efficiency and survival time (4).

Metabolic syndrome (MetS) is a set of complex metabolic disorder syndromes, which describes a pathologic state in protein, fat, carbohydrate, and other metabolic components. The main causes of MetS are obesity (especially centripetal obesity) and insulin resistance. With an increase in the number of obese patients worldwide, the MetS population has also raised (5). Multiple studies have indicated that MetS and related indicators such as obesity, hyperinsulinemia, insulin resistance, inflammation, and adipocytokine secretion disorders are associated with the occurrence, recurrence, and all-cause mortality of BC (6–8). Stebbing et al. confirmed that MetS before adjuvant chemotherapy (ACT) can predict poor clinical response of BC patients with metastasis (P = 0.030) (9). Moreover, studies found that insulin was relevant to the efficiency of BCNACT (10, 11). However, obesity (12, 13), diabetes, high fasting blood glucose (FBG) (14, 15), and blood lipid (16, 17) were not consistent in predicting the efficiency of BCNACT. Furthermore, the research on the relationship between MetS and the efficiency of BCNACT is still very limited. To this end, we evaluated the potential contribution of MetS and relevant indicators in predicting the response of BCNACT. The correlation between the two and the predict ability can be determined by the traditional statistical and machine learning (logistic regression) approaches. In addition, studies have found that, in the estrogen receptor (ER) (+) subgroup, blood glucose (18) and lipid (17) are related to the efficacy of BC chemotherapy; therefore, we also investigated this relationship under different ER states.

MetS was observed to have hyperuricemia and vascular endothelial dysfunction, and vascular endothelial dysfunction could lead to microalbuminuria and mild renal injury (19). Furthermore, oxidative stress was considered as a pathogenesis mechanism of MetS. Lactate dehydrogenase (LDH) was observed to induce conditions of oxidative stress (20). Oxidative stress could disrupt the secretion of adipocytokines (adipose-derived hormones) including adiponectin, plasminogen activator inhibitor 1, interleukin-6, and monocyte chemoattractant protein 1 (21). These adipocytokines mediated the development of MetS by participating in the regulation of insulin sensitivity and glucose metabolism (22); thus, MetS might be accompanied by low-grade inflammatory reaction. Therefore, apart from MetS and its components, uric acid (UA), creatinine (Cr), LDH, neutrophil-lymphocyte ratio (NLR), lymphocyte-monocyte ratio (LMR), and platelet-lymphocyte ratio (PLR) were also considered as potential predictors of BCNACT. In addition, studies have found that, in the postmenopausal subgroup, MetS is related to stage and lymph node metastasis of BC patients, so we also studied the relationship between MetS and clinical characteristics of BC under different menstrual states (23).

## Patients and Methods

### Population Study

**Figure 1:**
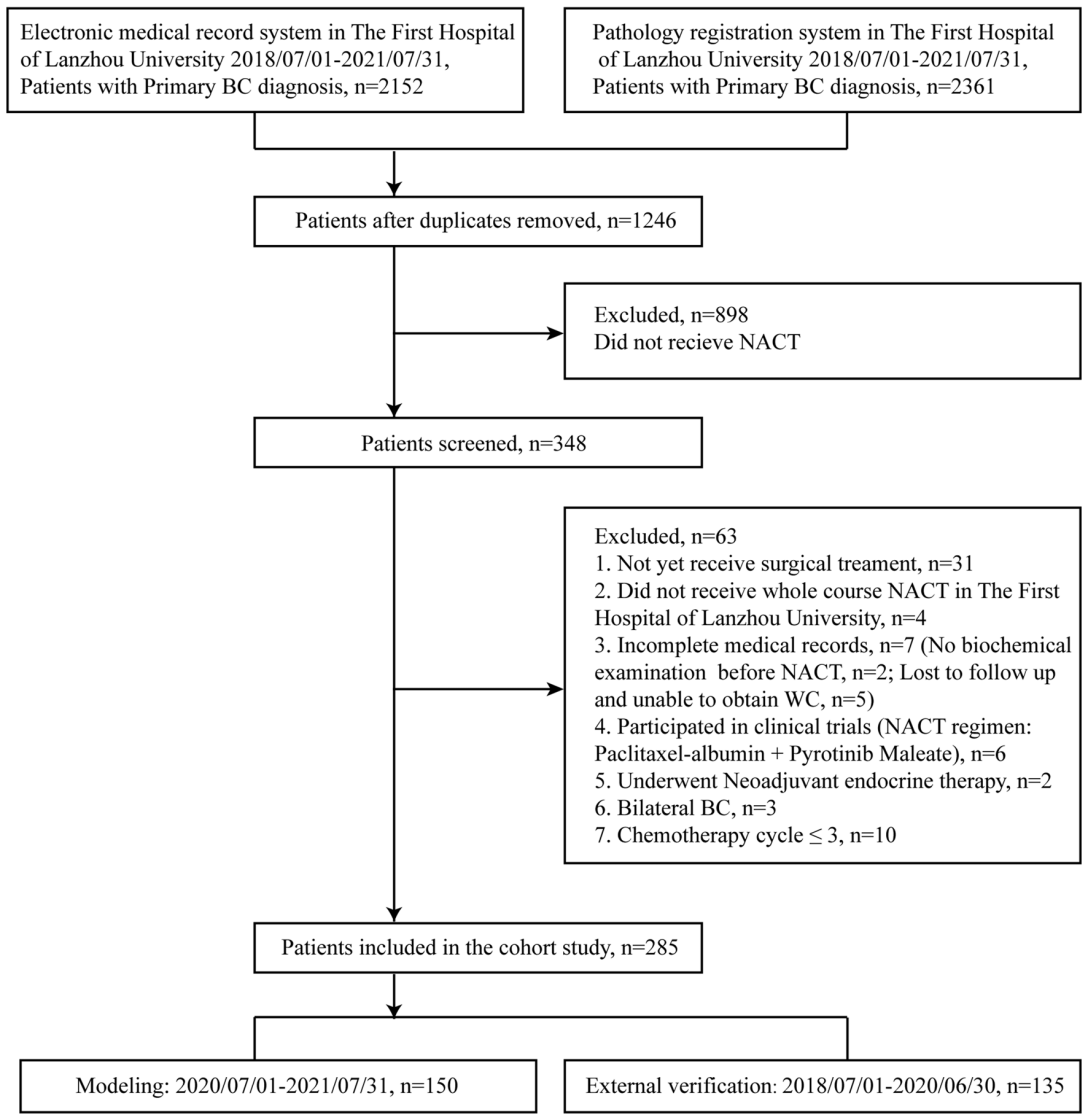
Selection of patients for present study. BC, breast cancer; NACT, neoadjuvant chemotherapy; WC, waist circumference.

In this study, patients diagnosed with BC in The First Hospital of Lanzhou University between 1 July, 2018 and 31 July, 2021 were retrieved through the electronic medical record system (n = 2,152) and the pathologic registration system (n = 2,361). A total of 1,246 patients were retained after duplicates were removed, and 348 of them underwent NACT. Patients who not yet received surgery (n = 31) did not receive whole course NACT in The First Hospital of Lanzhou University (n = 4), had no biochemical experiment (n = 2), lost to follow up and unable to obtain waist circumference (WC) (n = 5), participated in clinical trials (NACT regimen: albumin paclitaxel + perlotinib maleate) (n = 6), and underwent neoadjuvant endocrine therapy (n = 2). Patients with bilateral BC (n = 3) or whose chemotherapy cycle ≤3 (n = 10) were excluded. Finally, a total of 285 primary BC patients were evaluated. Study model was built with 150 patients (1 July, 2020 to 31 July, 2021), and 135 patients (1 July, 2018 to 30 June, 2020) were used for external validation (**Figure 1**). Patients included in this study did not receive any antitumor treatment before diagnosis and underwent surgery after four to eight cycles of standard NACT. They had no infectious diseases, hematological diseases, or severe liver or kidney dysfunction and did not take glucocorticoids and other drugs that might affect laboratory indicators within 3 months before diagnosis. The study was approved by the ethics committee of The First Hospital of Lanzhou University (No. LDYYLL2021-265). Written informed consent has been remitted in this study.

### Medical Record Collection

Clinical data were obtained through the electronic medical record system of The First Hospital of Lanzhou University and *via* telephone follow up. WC was measured at the navel level (24). The weight and height were measured using a digital scale while patients were not wearing heavy clothes and shoes. Body mass index (BMI) was calculated according to the standard formula of weight (kg)/height (m^2^). Blood pressure was measured using the same electronic sphygmomanometer. Blood and biochemical indicators were tested through blood samples from patients within 2 weeks before NACT. All patients underwent clinical staging through breast ultrasound, computed tomography (CT) scans or magnetic resonance imaging (MRI) before NACT. Ultrasound-guided breast puncture (lymph node puncture if necessary) was also performed in patients to clarify the pathology type and molecular classification of BC. Pathological data were independently evaluated by two experienced pathologists from The First Hospital of Lanzhou University. If the results of the two pathologists were inconsistent, a second evaluation was conducted until reaching a consensus.

### MetS Definition

The, 2006 criteria of IDF (International Diabetes Federation) were adopted to diagnose MetS (5, 25). For a person to be defined as having MetS, they must have WC >80 cm, with the presence of two or more of the following conditions: 1. FBG > 5.6 mmol/L (100 mg/dl) or diagnosed with diabetes; 2. high-density lipoprotein cholesterol (HDL-C) <1.3 mmol/L (50 mg/dl) or drug therapy for low HDL-C; 3. blood triglycerides (TG) >1.7 mmol/L (150 mg/dl) or undergoing medical treatment for elevated TG; 4. blood pressure >130/85 mmHg or drug treatment for hypertension.

### Treatment

Patients with human epidermal growth factor 2 (HER2) (−) received AC-T, AC, or TAC regimens. Most of patients with HER2 (+) received TCbHP, THP, AC-TH, TCbH, AC-THP, or TH regimens (T: taxane, including docetaxel, albumin paclitaxel or paclitaxel; A: anthracycline, C: cyclophosphamide; including epirubicin, pyridoxorubicin or doxorubicin; Cb: carboplatin; H: trastuzumab; P: pertuzumab). The NACT protocol used for the patients is shown in **Supplementary Tables 1**, **2**. It was reported that taxanes can improve the response of BCNACT (26), so the chemotherapy regimens were grouped into categories that either included or excluded taxanes (12). The chemotherapy dose was provided according to the body surface area, and an individualized treatment plan was formulated according to the Chinese Society of Clinical Oncology guidelines and patient’s conditions.

### NACT Response Evaluation

Pathologic complete response (pCR) was defined as no residual cancer lesions in any excised breast tissue or lymph nodes. Clinical response was evaluated according to the response evaluation criteria in solid tumors criteria (RECIST) version 1.1 (27). Partial response (PR) and complete response (CR) were defined as a good clinical response; progressive disease (PD) and stable lesions (SD) were defined as a poor clinical response. Pathologic response was assessed according to the Miller and Payne grading (MP grading) (28, 29). G1–G3 were defined as poor pathologic response and G4–G5 were defined as good pathologic response (30).

### Statistical Analysis

The SPSS 26.0 software was utilized to conduct statistical analysis, and the GraphPad Prism 9 and R 4.1.2 software were used to draw pictures. Dichotomous variables were defined by the optimal cutoff value of the receiver operating characteristic curve (ROC). Measurement data were analyzed by *t*-test and counting data were analyzed by Chi-square (χ2) test, Fisher’s exact test, or non-parametric test. Univariate, multivariate logistics regression and subgroup (log-linear regression) analyses were made to assess possible predictors on pCR, clinical, and pathologic response. Predictors related to MetS [age at initiation of treatment, BMI, menstrual status, WC, FBG, systolic blood pressure (SBP), diastolic blood pressure (DBP), TG, HDL-C, and UA] and predictors with P < 0.05 on univariate analysis were included in multivariate analysis. The confidence interval (CI) of the risk ratio (RR) is 95% and p-value < 0.05 is considered statistically significant.

## Results

### Patient Characteristics

This study included 104 non-MetS (69.30%) and 46 MetS patients (30.70%). The average age was 49.43 ± 10.314 years old (26–76 years old). The average chemotherapy cycle was 6.69 ± 1.589. BCNACT scheme was shown in **Supplementary Table 1**. The age, BMI, WC, FBG, SBP, DBP, TG, and UA levels of MetS patients were significantly higher than those without MetS, and the HDL-C level was significantly lower than those without MetS. Postmenopausal patients were more prone to have MetS. These two groups were comparable in tumor size, lymph node status, stage, histological type, NACT regimen, molecular subtype, ER, progesterone receptor (PR), HER2, and Ki-67 expression levels (**Table 1**). In addition, regardless of menstrual status, the status of MetS was not related to clinical characteristics (**Supplementary Table 3**).

**Table 1:** Population and clinicopathologic characteristics.

Characteristics	Non-MetS (%/ ± SD)	MetS (%/ ± SD)	Total	p-value
	104(69.30%)	46(30.70%)	150	
Mean age, year				
<50	52	13	65	**0.010**
≥50	52	33	85	
BMI, kg/m^2^				
<25	78	16	94	**<0.001**
25<T ≤ 30	25	24	49	
T>30	1	6	7	
Menopausal status				
Premenopausal	59	17	76	**0.020**
Postmenopausal	45	29	74	
WC, cm				
≤80	65	0	65	**<0.001**
>80	39	46	85	
FBG, mmol/L				
≤5.6	91	23	114	**<0.001**
>5.6	13	23	36	
Blood pressure				
SBP, mmHg				
≤130	65	19	84	**0.013**
>13	39	27	66	
DBP, mmHg				
≤85	78	26	104	**0.020**
>85	26	20	46	
Lipid profile				
TG, mmol/L				
≤1.7	89	22	111	**<0.001**
>1.7	15	24	39	
HDL-C, mmol/L				
<1.3	60	8	68	**<0.001**
≥ 1.3	44	38	82	
TC, mmol/L	4.46 ± 0.93	4.60 ± 1.07		0.436
LDL-C, mmol/L	2.92 ± 0.74	3.07 ± 0.82		0.268
UA, μmol/L	261.02 ± 60.56	287.48 ± 79.62		**0.027**
Cr, μmol/L	57.88 ± 8.04	57.70 ± 8.86		0.905
LDH, U/L	179.66 ± 38.74	186.20 ± 43.53		0.361
Inflammation				
NLR	3.07 ± 2.11	3.13 ± 2.86		0.900
LMR	7.89 ± 14.99	6.30 ± 2.92		0.476
PLR	168.02 ± 86.31	145.00 ± 62.23		0.105
Tumor size				0.070
T ≤ 2 cm	19	5	24	
2 cm<T ≤ 5 cm	78	35	113	
T>5 cm	7	6	13	
Lymph node				0.271
Negative	34	12	46	
Positive	70	34	104	
Clinical stage				0.065
I	4	0	4	
IIA	44	16	60	
IIB	49	25	74	
III	7	5	12	
Pathogenic type				0.338
Invasive carcinoma	71	31	102	
Invasive carcinoma with ductal carcinoma	28	10	38	
Others	5	5	10	
Molecular subtype				0.383
HER2+/HR+	19	10	29	
HER2+/HR−	31	8	39	
Luminal	43	24	67	
TNBC	11	4	15	
ER				0.461
Negative	41	17	58	
Positive	63	29	92	
PR				0.462
Negative	59	25	84	
Positive	45	21	66	
HER2				0.202
Negative	54	28	82	
Positive	50	18	68	
Ki-67				0.072
Negative	2	4	6	
Positive	102	42	144	
TNBC				0.489
Yes	11	4	15	
No	93	42	135	
NACT regimen				0.281
Non-taxane based	5	4	9	
Taxane based	99	42	141	

MetS, metabolic syndrome; SD, standard deviation; BMI, body mass index; WC, waist circumference; FBG, fasting blood glucose; SBP, systolic blood pressure; DBP, diastolic blood pressure; TG, triglycerides; HDL-C, high-density lipoprotein cholesterol; TC, total cholesterol; LDL-C; low-density lipoprotein cholesterol; UA, uric acid; Cr, creatinine; LDH, lactate dehydrogenase; NLR, neutrophil-lymphocyte ratio; LMR, lymphocyte-monocyte ratio; PLR, platelet-lymphocyte ratio; HER2, human epidermal growth factor 2; HR, hormone receptor; TNBC, triple negative breast cancer; ER, estrogen receptor; PR, progesterone receptor; NACT, neoadjuvant chemotherapy. Bold means p-value < 0.05.

### Relationship Between MetS and BCNACT Response

**Figure 2:**
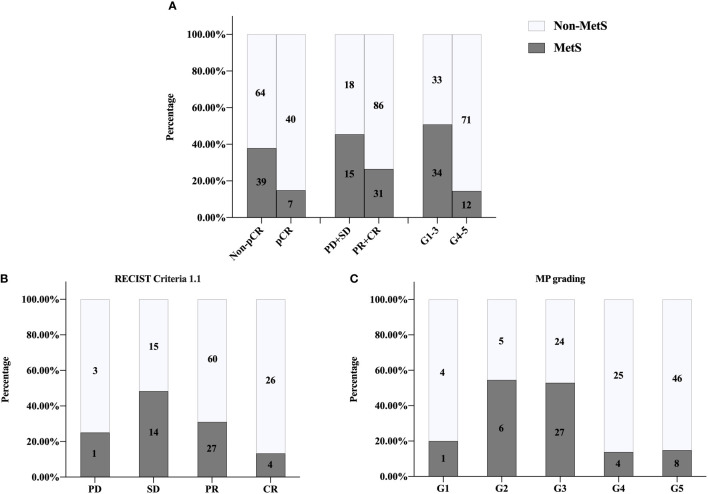
Univariate analysis of relationship between MetS and NACT response. **(A)** pCR, P = 0.003; clinical responses, P = 0.033; pathologic responses, P <0.001. **(B)** RECIST1.1 criteria P = 0.004. **(C)** MP grading P < 0.001. MetS, metabolic syndrome; pCR, pathologic complete response; PD, progressive disease; SD, stable lesions; PR, partial response; CR, complete response; MP, Miller-Payne; RECIST, response evaluation criteria in solid tumors.

The overall pCR rate of BCNACT was 31.33%, of which the pCR rate of HER2 (+) patients was 55.88% and that of HER2 (−) patients was 10.98%. Compared with non-MetS patients, MetS patients had lower pCR rate (P = 0.003), poorer clinical response (P = 0.033), and poorer pathological response (P < 0.001) (**Figure 2A**). Mass shrinkage (RECIST criteria 1.1) was more obvious (P = 0.004) (**Figure 2B**), and the pathological grade was lower (P < 0.001) **(** **Figure 2C**) in non-MetS patients.

**Figure 3:**
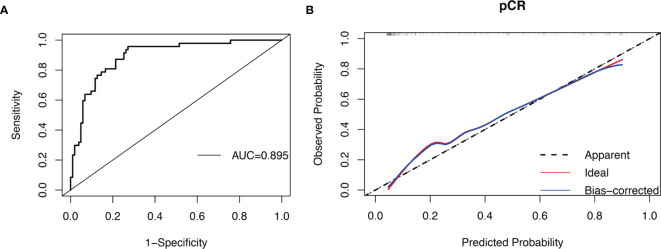
ROC curve and calibration curve of BCNACT pCR prediction model: **(A)** ROC curve. **(B)** calibration curve.

When taking pCR as the outcome, multivariate analysis found that non-MetS patients had a higher probability of pCR (P = 0.003) (**Table 2**). According to ROC curve, the C index of this model was 0.895 (95% CI, 0.841–0.948; P < 0.001), and the sensitivity and specificity were 0.957 and 0.728 respectively (**Figure 3A**). The calibration curve shows that the predicted probability of the model is in good agreement with ideal probability (**Figure 3B**).

**Table 2:** Univariate and multivariate analysis of laboratory and clinical indicators with BCNACT pathologic complete response.

Indicators	Total	pCR		Univariate analysis	Multivariate analysis (Hosmer–Lemeshow test, P = 0.250)	
		No	Yes	p-value	RR (95% CI)	p-value
	150	103	47			
Age, year				**0.018**		0.064
<50	65	51	14		1 (reference)	
≥50	85	52	33		0.278 (0.072–1.076)	
BMI, kg/m^2^				0.420		0.357
<25	94	66	28		1 (reference)	
25 ≤BMI <30	49	30	19		4.189*10^8^ (0.000−~)	0.999
≥30	7	7	0		1.010*10^9^ (0.000−~)	0.999
Menopausal status				0.122		0.541
Premenopausal	76	56	20		1 (reference)	
Postmenopausal	74	47	27		1.511 (0.403–5.672)	
WC, cm				0.204		0.539
≤93.665	131	92	39		1 (reference)	
>93.665	19	11	8		0.596 (0.115–3.102)	
FBG, mmol/L				0.097		0.059
≤5.415	102	74	28		1 (reference)	
>5.415	48	29	19		0.312 (0.103–1.046)	
Blood pressure						
SBP, mmHg				0.060		0.470
≤137.5	95	70	25		1 (reference)	
>137.5	55	33	22		0.617 (0.166–2.289)	
DBP, mmHg				0.233		0.897
≤83.5	91	65	26		1 (reference)	
>83.5	59	38	21		1.087 (0.304–3.887)	
Lipid profile						
TG, mmol/L				0.242		0.868
≤1.525	97	69	28		1 (reference)	
>1.525	53	34	19		1.107 (0.332–3.687)	
HDL-C, mmol/L				**0.027**		0.980
≤1.465	121	88	33		1 (reference)	
>1.465	29	15	14		0.984 (0.296–3.274)	
TC, mmol/L				**0.021**		0.786
≤4.720	90	68	22		1 (reference)	
>4.720	60	35	25		0.665 (0.035–12.761)	
LDL-C, mmol/L				**0.047**		0.822
≤3.225	93	69	24		1 (reference)	
>3.225	57	34	23		1.419 (0.067–29.963)	
UA, μmol/L				0.234		0.442
≤221.5	36	27	9		1 (reference)	
>221.5	114	76	38		1.613 (0.477–5.451)	
Cr, μmol/L				0.169		
≤53.9	48	36	12			
>53.9	102	67	35			
LDH, U/L				0.151		
≤162	52	39	13			
>162	98	64	34			
Inflammation						
NLR				0.142		
≤1.925	49	37	12			
>1.925	101	66	35			
LMR				0.320		
≤6.585	95	67	28			
>6.585	55	36	19			
PLR				0.065		
≤114.085	39	31	8			
>114.085	111	72	39			
Tumor size				0.327		
T ≤ 2 cm	24	16	8			
2 cm <T ≤ 5 cm	113	77	36			
T > 5 cm	13	10	3			
Lymph node				0.336		
Negative	46	30	16			
Positive	104	73	31			
Clinical stage				0.252		
I	4	1	3			
IIA	60	42	18			
IIB	74	51	23			
III	12	9	3			
Pathogenic type				**0.014**		0.941
Invasive carcinoma	102	172	30		1 (reference)	
Invasive carcinoma with ductal carcinoma	38	21	17		8.763*10^8^ (0.000−~)	0.999
Others	10	10	0		1.061*10^9^ (0.000-−~)	0.999
Molecular subtype				**<0.001**		
HER2+/HR+	29	15	14			
HER2+/HR−	39	16	23			
Luminal	67	61	6			
TNBC	15	11	4			
ER				**0.004**		0.610
Negative	58	32	26		1 (reference)	
Positive	92	71	21		0.716 (0.198–2.584)	
PR				**0.002**		0.096
Negative	84	49	35		1 (reference)	
Positive	66	54	12		3.177 (0.814–12.398)	
HER2				**<0.001**		**<0.001**
Negative	82	73	9		1 (reference)	
Positive	68	30	38		0.123 (0.038–0.396)	
Ki-67				0.278		
Negative	6	3	3			
Positive	144	100	44			
TNBC				0.465		
Yes	15	11	4			
No	135	92	43			
NACT regimen				0.165		
Non-taxane based	9	8	1			
Taxane based	141	95	46			
MetS				**0.003**		**0.003**
No	104	64	40		1 (reference)	
Yes	46	39	7		10.765 (2.256–51.361)	

pCR, pathologic complete response; RR, risk ratio; CI, confidence interval; BMI, body mass index; WC, waist circumference; FBG, fasting blood glucose; SBP, systolic blood pressure; DBP, diastolic blood pressure; TG, triglycerides; HDL-C, high-density lipoprotein cholesterol; TC, total cholesterol; LDL-C; low-density lipoprotein cholesterol; UA, uric acid; Cr, creatinine; LDH, lactate dehydrogenase; NLR, neutrophil-lymphocyte ratio; LMR, lymphocyte-monocyte ratio; PLR, platelet-lymphocyte ratio; HER2, human epidermal growth factor 2; HR, hormone receptor; TNBC, triple negative breast cancer; ER, estrogen receptor; PR, progesterone receptor; NACT, neoadjuvant chemotherapy. Bold means p-value < 0.05. * means multiplication.

When taking clinical remission as the outcome, multivariate analysis found that, compared with patients with BMI ≥25 and <30 kg/m^2^, patients with BMI <25 kg/m^2^ were less likely to have good clinical response (P = 0.006). Compared with patients with TG ≤1.3, patients with TG >1.3 were less likely to obtain good clinical response (P = 0.036) (**Supplementary Table 4**). When pathological remission was used as the outcome, multivariate analysis found that patients with TG >0.865 were less likely to obtain clinical remission than patients with TG ≤0.865 (P = 0.007) (**Supplementary Table 5**). MetS was not found to be associated with clinical and pathological response of BCNACT. Analysis according to ER status found that the relationship between fast blood glucose, serum lipid, and BCNACT response was not affected by ER expression status (**Supplementary Table 6**).

### Subgroup Analysis of MetS and BCNACT pCR

**Figure 4:**
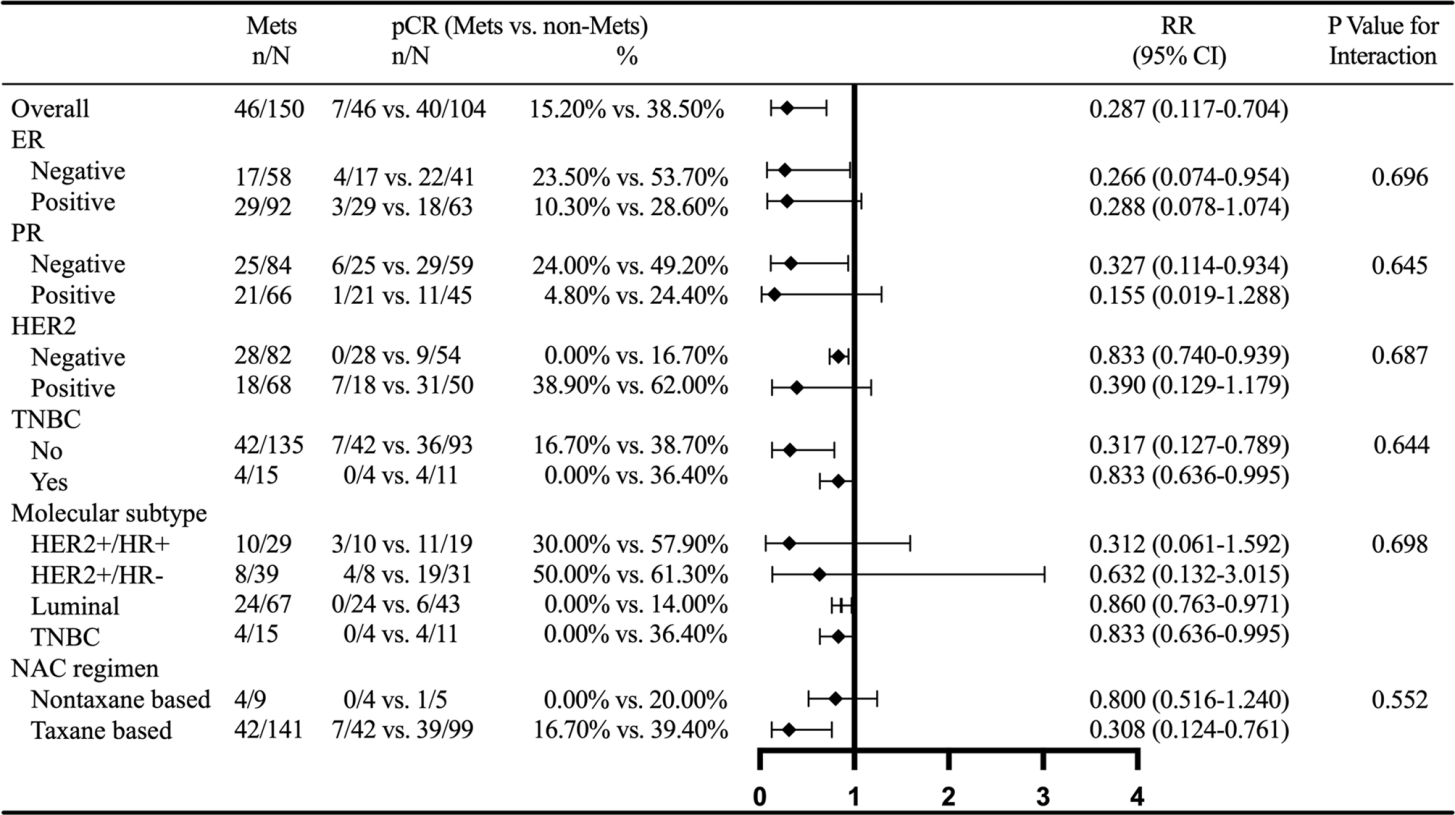
Subgroup analysis of MetS and BCNACT pCR. MetS, metabolic syndrome; pCR, pathologic complete response; RR, risk ratio; CI, confidence interval; ER, estrogen receptor; PR, progesterone receptor; HER2, human epidermal growth factor 2; TNBC, triple negative breast cancer; NACT, neoadjuvant chemotherapy.

Interaction analysis showed that MetS had no interaction with ER, PR, HER2, triple negative BC (TNBC), molecular subtype, and NACT regimen. Subgroup analysis showed that the relationship between MetS and BCNACT pCR was more significant in ER (−), PR (−), HER2 (−), TNBC (−), TNBC, luminal subgroup, and NACT regimen based on taxane (**Figure 4**).

### External Validation of Relationship Between MetS and BCNACT Response

**Figure 5:**
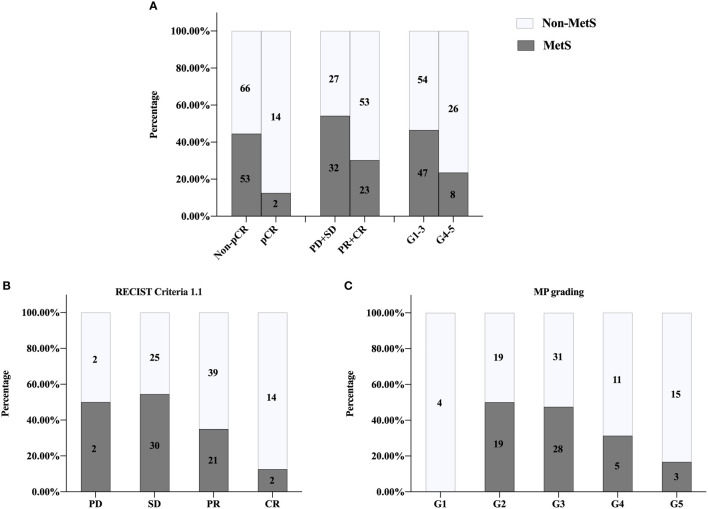
External validation of univariate analysis on relationship between MetS and NACT response. **(A)** pCR, P = 0.011; clinical responses, P = 0.004; pathologic responses, P = 0.014. **(B)** RECIST1.1 criteria P < 0.001. **(C)** MP grading P = 0.048. MetS, metabolic syndrome; pCR, pathologic complete response; PD, progressive disease; SD, stable lesions; PR, partial response; CR, complete response; MP, Miller-Payne; RECIST, response evaluation criteria in solid tumors.

Patient characteristics of external validation group were showed in **Supplementary Table 7**. There was no relationship between MetS and clinical characteristics of BC patients regardless of menstrual status (**Supplementary Table 8**). BCNACT scheme was shown in **Supplementary Table 2**. Univariate analysis confirmed that MetS was associated with BCNACT response (pCR, P = 0.011; clinical response, P = 0.004; pathological response; P = 0.014; RECIST criteria 1.1, P < 0.001; MP grade, P = 0.048) (**Figure 5**).

**Figure 6:**
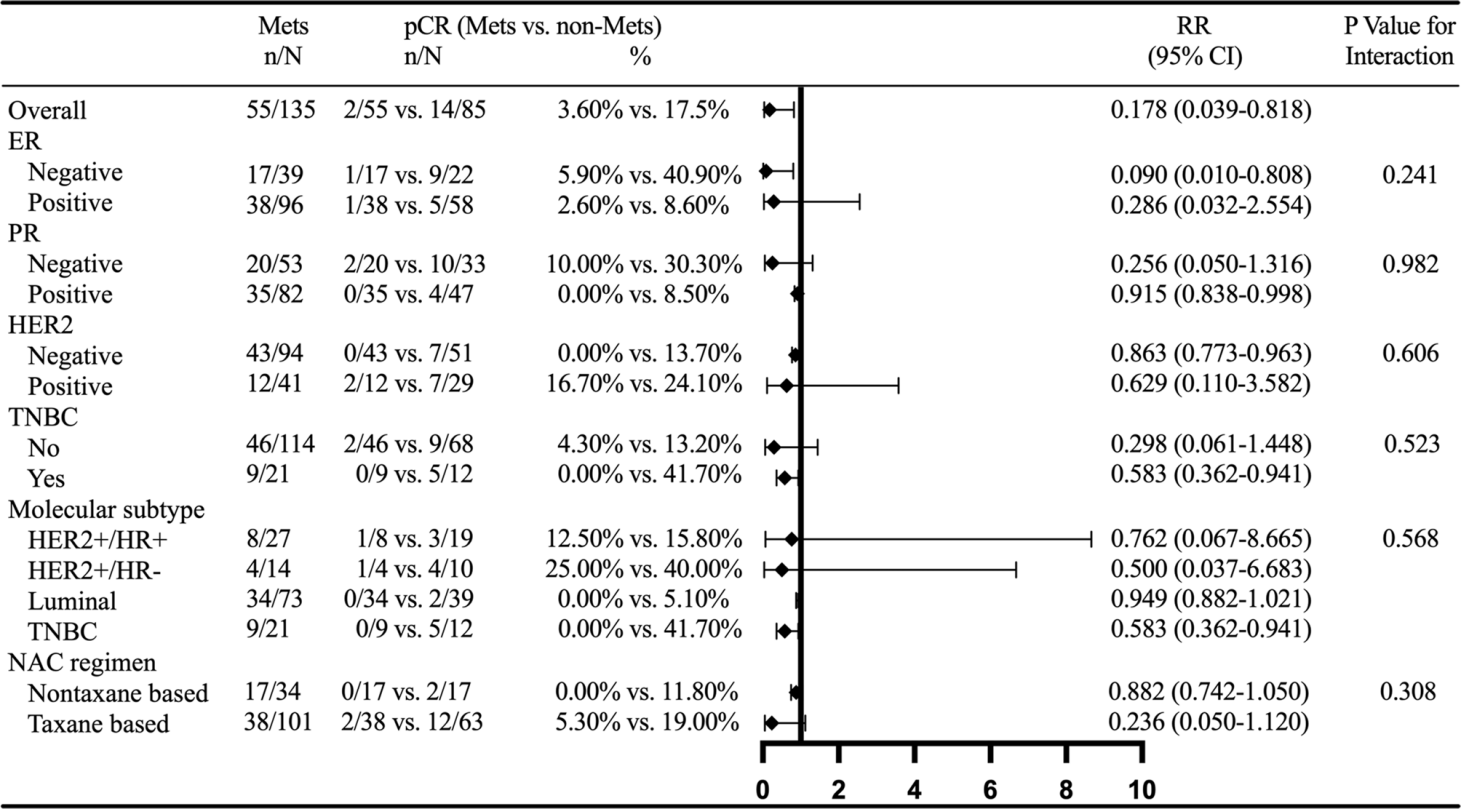
External validation of subgroup analysis on the relationship between MetS and BCNACT pCR. MetS, metabolic syndrome; pCR, pathologic complete response; RR, risk ratio; CI, confidence interval; ER, estrogen receptor; PR, progesterone receptor; HER2, human epidermal growth factor 2; TNBC, triple negative breast cancer; NACT, neoadjuvant chemotherapy.

Multivariate analysis confirmed that MetS was associated with BCNACT pCR (P = 0.046). Multivariate analysis also found that patients with FBG ≤5.415 were more likely to get obtain pCR than patients with FBG >5.415 (P = 0.023) (**Table 3**). The C index of the model was 0.917 (95% CI, 0.862–0.973; P < 0.001), and the sensitivity and specificity were 0.983 and 0.714, respectively (**Supplementary Figure 1**). Multivariate analysis did not find indicators related to clinical and pathological response (**Supplementary Tables 9, 10**). Subgroup analysis confirmed that the relationship between MetS and BCNACT pCR was more significant in ER (−), HER2 (−), and TNBC subgroups (**Figure 6**). It was not found that the relationship between blood lipid, blood glucose, and response was affected by ER expression status (**Supplementary Table 11**).

**Table 3:** Univariate and multivariate analyses of laboratory and clinical indicators with BCNACT pathologic complete response on external validation patients.

Indicators	Total	pCR		Univariate analysis	Multivariate analysis (Hosmer–Lemeshow test, P = 0.853)	
		No	Yes	p-value	RR (95% CI)	p-value
	135	119	16			
Age, year				0.517		0.226
<50	64	56	8		1 (reference)	
≥50	71	63	8		0.277 (0.035–2.207)	
BMI, kg/m^2^				0.435		0.259
<25	83	73	10		1 (reference)	
25 ≤ BMI <30	44	38	6		3.388*10^6^ (0.000−~)	0.999
≥30	8	8	0		1.615*10^7^ (0.000−~)	0.999
Menopausal status				0.450		0.092
Premenopausal	78	68	10		1 (reference)	
Postmenopausal	57	51	6		6.419 (0.737–55.916)	
WC, cm				**0.048**		0.998
≤93.665	113	97	16		1 (reference)	
>93.665	22	22	0		3.883*10^8^ (0.000−~)	
FBG, mmol/L				**0.012**		**0.023**
≤5.415	91	76	15		1 (reference)	
>5.415	44	43	1		79.074 (1.809–3456.590)	
Blood pressure						
SBP, mmHg				0.430		0.060
≤137.5(没变)	99	88	11		1 (reference)	
>137.5	36	31	5		0.038 (0.002–1.093)	
DBP, mmHg				0.262		0.603
≤83.5	96	83	13		1 (reference)	
>83.5	39	36	3		1.788(0.200–15.958)	
Lipid profile						
TG, mmol/L				0.240		0.666
≤1.525	95	82	13		1 (reference)	
>1.525	40	37	3		1.528 (0.223–10.452)	
HDL-C, mmol/L				0.337		0.997
≤1.465	27	25	2		1 (reference)	
>1.465	108	94	14		0.996 (0.111–8.955)	
TC, mmol/L				0.195		
≤4.720	75	64	11			
>4.720	60	55	5			
LDL-C, mmol/L				0.467		
≤3.225	87	76	11			
>3.225	48	43	5			
UA, μmol/L				0.295		0.081
≤221.5	39	33	6		1 (reference)	
>221.5	96	86	10		4.788 (0.827–27.734)	
Cr, μmol/L				0.567		
≤53.9	28	25	3			
>53.9	107	94	13			
LDH, U/L				0.142		
≤162	36	34	2			
>162	99	85	14			
Inflammation						
NLR				0.240		
≤1.925	40	37	3			
>1.925	95	82	13			
LMR				0.309		
≤6.585	98	85	13			
>6.585	37	34	3			
PLR				0.415		
≤114.085	33	30	3			
>114.085	102	89	13			
Tumor size				0.346		
T ≤ 2 cm	25	22	3			
2 cm < T≤ 5 cm	100	87	13			
T > 5 cm	10	10	0			
Lymph node				0.102		
Negative	52	43	9			
Positive	83	76	7			
Clinical stage				0.076		
I	10	8	2			
IIA	57	49	8			
IIB	55	49	6			
III	13	13	0			
Pathogenic type				1.000		
Invasive carcinoma	125	109	16			
Invasive carcinoma with ductal carcinoma	6	6	0			
Others	0	8	8			
Molecular subtype				**<0.001**		
HER2+/HR+	27	23	4			
HER2+/HR–	14	9	5			
Luminal	73	71	2			
TNBC	21	16	5			
ER				**0.003**		0.552
Negative	39	29	10		1 (reference)	
Positive	96	90	6		1.764 (0.272–11.413)	
PR				**0.002**		0.093
Negative	53	41	12		1 (reference)	
Positive	82	78	4		7.208 (0.719–72.297)	
HER2				**0.020**		**0.023**
Negative	94	87	7		1 (reference)	
Positive	41	32	9		0.157 (0.063–0.519)	
Ki-67				0.219		
Negative	14	11	3			
Positive	121	108	13			
TNBC				0.077		
Yes	21	16	5			
No	114	103	11			
NACT regimen				0.176		
Non-taxane based	34	32	2			
Taxane based	101	87	14			
MetS				**0.011**		**0.046**
No	80	66	14		1 (reference)	
Yes	55	53	2		9.416 (1.038–85.443)	

pCR, pathologic complete response; RR, risk ratio; CI, confidence interval; BMI, body mass index; WC, waist circumference; FBG, fasting blood glucose; SBP, systolic blood pressure; DBP, diastolic blood pressure; TG, triglycerides; HDL-C, high-density lipoprotein cholesterol; TC, total cholesterol; LDL-C; low-density lipoprotein cholesterol; UA, uric acid; Cr, creatinine; LDH, lactate dehydrogenase; NLR, neutrophil-lymphocyte ratio; LMR, lymphocyte-monocyte ratio; PLR, platelet-lymphocyte ratio; HER2, human epidermal growth factor 2; HR, hormone receptor; TNBC, triple negative breast cancer; ER, estrogen receptor; PR, progesterone receptor; NACT, neoadjuvant chemotherapy. Bold means p-value < 0.05. * means multiplication.

## Discussion

NACT is an indispensable treatment for locally advanced BC, which can shrink the tumor volume, increase the opportunity of operation and breast preservation, lessen the surgical trauma, eliminate the minor subclinical cancer focus, reduce the activity of tumor cells, so as to decrease the risk of distant metastasis, and provide the basis of drug sensitivity for preoperative ACT. Furthermore, predicting the response of BCNACT is helpful to evaluate the prognosis of patients, promote individualized treatment, and further improve the BCNACT response rate. To the best of our knowledge, this study is the first research, which specifically explores the relationship between the response of BCNACT and MetS. Previously, only the relationship between MetS components and BCNACT response was studied. MetS can be diagnosed merely through routine examination, without the need for new technology or equipment. Thus, it has the advantages of low cost, high efficiency, and ease to generalize in predicting the efficiency of BCNACT. Studies have shown that MetS is associated with later staging (P = 0.022) and lymph node metastasis (P = 0.028) in postmenopausal BC (23). Our study did not find this link (**Supplementary Tables 3**, **8**), which may be due to the different clinical characteristics of the population participating in BCNACT from the whole BC population with postmenopausal. MetS patients are also accompanied by hyperuricemia, persistent low-grade inflammatory reaction, oxidative stress, and mild renal injury. Our study found that MetS patients had higher UA levels (**Table 1**), which was consistent with other study (31). In addition, external validation found higher LDH levels in MetS patients (**Supplementary Table 7**), suggesting that MetS patients were easily predisposed to oxidative stress than non-MetS patients. This research has not yet found a direct link between MetS and mild renal injury (Cr) or persistent low inflammatory reaction (NLR, LMR, and PLR) (**Table 1** and **Supplementary Table 7**), perhaps MetS did not participate in the pathogenesis of BC through these mechanisms.

In our study, univariate analysis found that patients with MetS before chemotherapy were more difficult to achieve pCR, clinical, and pathological response than non-MetS (**Figures 2**, **5**). Multivariate analysis found that MetS was associated with BCNACT pCR (**Tables 2**, **3**). A study demonstrated that, among BC patients with metastasis, non-MetS patients before ACT were easier to achieve clinical response than MetS patients (9); this study is a good complement in predicting the response of BC chemotherapy *via* MetS. Alan et al. did not find the relationship between MetS and BCNACT pCR in the study of 55 patients (33% vs. 23%, P = 0.200) (11), but the sample size of this study is small and the diagnostic criteria of MetS are different from this study (32).

Regarding the relationship between obesity and BCNACT response, studies have discovered that obese patients have a lower pCR rate (OR = 0.59; 95% CI, 0.37–0.95) (12), and similar studies have confirmed it. Maybe insufficient dose of obese patients led to poor response (33). However, a meta-analysis concluded that BMI is not associated with BCNACT response (34). Our study found that overweight patients were more likely to achieve clinical response than patients with normal weight (**Supplementary Table 4**). This conclusion is contrary to previous studies, and the external validation group did not find this association.

As for the relationship between serum lipids and BCNACT response, Hilvo et al. found that lower-level TG suggests BCNACT pCR (16). Our study found that low-level TG was easy to obtain better clinical and pathological response (**Supplementary Tables 4, 5**), but the external validation group did not confirm this relationship (**Supplementary Tables 9, 10**). A study has found that the lower HDL-C in the ER (+) subgroup suggests better clinical response (17), which may be due to, in different ER subgroups, the activation of different signal pathways during chemotherapy or tumor heterogeneity was different. However, our research did not discover an association between HDL-C and BCNACT response in ER (+) subgroup (**Supplementary Tables 6, 11**). In general, blood lipids can indicate the efficacy of BCNACT, but it is still necessary to use unified standards to further evaluate its predictive value in a larger scale.

With regard to the relationship between blood glucose and BCNACT response, Arici et al. suggested that diabetes and high FBG levels predicted poor pathological response (14). Alan et al. found that insulin resistance had an adverse relationship on BCNACT pCR (11). Our study did not find this relationship (**Table 2**), although external validation found higher FBG indicated lower PCR rate (**Table 3**). The study of Cao et al. also showed that FBG could not predict clinical response of BCNACT (15). Additionally, studies have found that hyperglycemia participated in ER (+) chemotherapy resistance. When hyperglycemia occurs, insulin-like growth factor 1 (IGF-1) concentration increases. IGF-1 can specifically induce FASN (fatty acid synthase) to activate the mitogen-activated protein kinase pathway of BC and increase ERα phosphorylation levels, which up-regulated nuclear localization of ERα. Nuclear ERα could raise the expression of CCND1 (cell cycle–related protein), which will weaken the inhibition of anticancer drugs on the proliferation of tumor cells (18, 35). However, our study did not consider that the relationship between blood glucose and BCNACT response related to ER status (**Supplementary Tables 6, 11**). In conclusion, we believe that this link between FBG and BCNACT response still needs to be verified in more rigorous research.

The relationship between MetS-related indicators UA, LDH, Cr, and BCNACT response was analyzed, but there is no predictor on response. Dennison et al. found that high LDH-B can predict BCNACT pCR on hormone receptor (HR) (+)/HER2 (−) (OR = 4.1, P < 0.001) and TNBC (OR = 3.0, P = 0.003) subtype (36). Therefore, it is necessary to further study the relationship among LDH subunits in various molecular subtypes and BCNACT response. In addition, studies have shown that NLR, LMR, and PLR predicted BCNACT response (37). However, our multivariate analysis did not confirm this conclusion, and the research conclusions of Alan et al. and Şahin et al. were consistent with ours (11, 38). In summary, we speculated that, as a comprehensive indicator, MetS could be a more precise indicator in predicting BCNACT response than MetS components. Multicenter studies are required to confirm the predictive role of blood lipids, blood glucose, and inflammatory parameters in BCNACT response.

In order to guide clinical practice better and verify the reliability of the conclusion, we conducted subgroup analysis on MetS and BCNACT pCR (**Figures 4**, **6**). The study of Tong et al. found that MetS was not indictor for BCNACT pCR in HER2 (+) (17.24% vs. 82.76%, P = 0.106) (39), which is consistent with our conclusion. However, we found significant differences in ER (−), HER2 (−), and TNBC subgroups. It is suggested that the intervention of MetS status in the above subgroups can improve BCNACT pCR rate more effectively.

This study has some limitations: 1. As a single-center study, only Chinese patients were included, the incidence of MetS varied greatly between China and other countries (40). Due to the small sample size and excessive fitting, reliable conclusions cannot be obtained *via* machine learning. The universality of conclusions from this study still needs to be verified in a larger population. 2. As a retrospective study, recall bias of patient data may exist. The diet and exercise status of patients before diagnosis had not been evaluated, which may be confounding factors (41). 3. This study did not rule out the interference of chemotherapy dose in the assessment of BCNACT response. During the NACT process, physicians appropriately adjusted the dose according to the degree of the patient’s tolerance or adverse reaction, and the dosages of some patients have also changed accordingly with the weight fluctuation. 4. In terms of protocol and dose, the guidelines referenced by external validation patients are not as good as those in modeling patients, which resulted in worse response than modeling group and cause inconsistencies on several secondary conclusions between modeling and external validation group.

Finally, our study suggests that it is necessary to conduct an in-depth study to find out the mechanism of BCNACT resistance in MetS patients, especially in ER (−), HER2 (−), and TNBC subgroups. Furthermore, a variety of methods to improve the metabolism of cancer patients can ameliorate the prognosis to a greater extent. For example, appropriate nutritional intervention and psychological support were observed to significantly prolong the survival rate for cancer patients (4). Heys et al. considered that supplementing l-arginine to BC patients could improve the NACT response (42). Adams et al. confirmed that resistance exercise training improved the life quality of BCNACT (43).

## Conclusion

MetS before NACT predicted BCNACT pCR, especially in ER (−), HER2 (−), and TNBC subgroups. Developing appropriate intervention strategies to rectify MetS status was speculated to improve the BCNACT response further.

## Data Availability Statement

The original contributions presented in the study are included in the article/**Supplementary Material**. Further inquiries can be directed to the corresponding author.

## Ethics Statement

The studies involving human participants were reviewed and approved by The First Hospital of Lanzhou University Ethics Committee. Written informed consent for participation was not required for this study in accordance with the national legislation and the institutional requirements.

## Author Contributions

WYZ and YL conceived the study. YL, PWX, NL, and FK designed the methods, analyzed the data and wrote the article. YL, PWX, AA, STY, and YLT collected the data. WY, PWX, NL, and FK revised the article. All authors contributed to the article and approved the submitted version.

## Conflict of Interest

The authors declare that the research was conducted in the absence of any commercial or financial relationships that could be construed as a potential conflict of interest.

## Publisher’s Note

All claims expressed in this article are solely those of the authors and do not necessarily represent those of their affiliated organizations, or those of the publisher, the editors and the reviewers. Any product that may be evaluated in this article, or claim that may be made by its manufacturer, is not guaranteed or endorsed by the publisher.
