# Supplementary material for: Metabolic Syndrome Predicts Response to Neoadjuvant Chemotherapy in Breast Cancer
Source: Front Oncol. 2022 Jul 1;12:899335. doi: 10.3389/fonc.2022.899335 (PMC9284232; doi:10.3389/fonc.2022.899335)
Supplement: Supplementary file 1 [file DataSheet_1.docx]

Supplementary Material

# Supplementary Figures and Tables

## Supplementary Figures


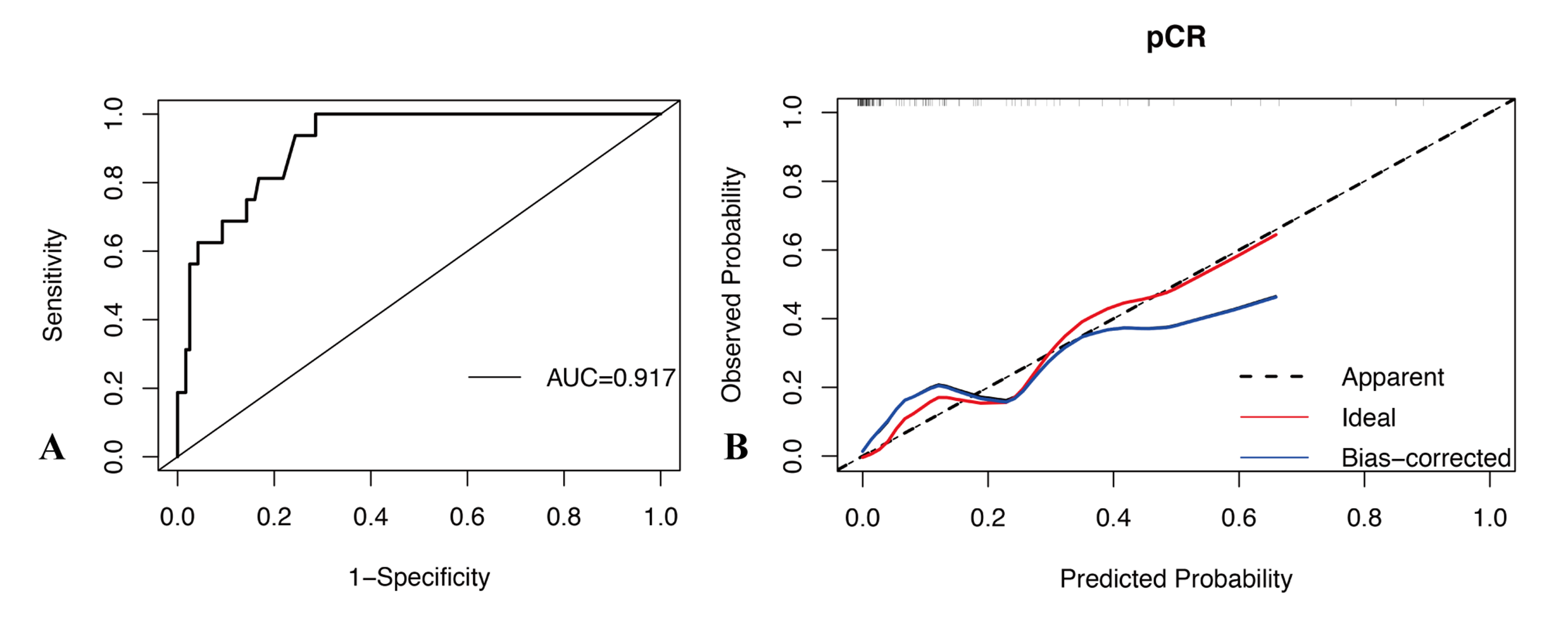


**Supplementary Figure 1.** External validation of ROC curve and calibration curve on BCNACT pCR prediction model: **(A)** ROC curve. **(B)** calibration curve.

## Supplementary Tables

**Supplementary Table 1.** NACT protocol for modeling patients.

| NACT regimen | AC-T | AC | TAC | TCbHP | THP | AC-TH | TCbH | AC-THP |
| --- | --- | --- | --- | --- | --- | --- | --- | --- |
| N (Total=142) | 58 | 10 | 9 | 37 | 20 | 4 | 3 | 1 |
| Patients with protocol change (n=8) | | | AC (one cycle) changed to TAC (four cycles)  AC (four cycles) changed to TCb (two cycles)  AC (four cycles) changed to TCb (four cycles)  AC (four cycles)-T (one cycle) changed to TCb (there cycles)  TCb (one cycle) changed to TAC (four cycles)  TCbHP (one cycle) changed to THP (five cycles)  TCbHP (four cycles) changed to THP (two cycles)  TH (five cycles) changed to THP (there cycles) | | | | | |

Abbreviations: T: taxane; A: anthracycline; C: cyclophosphamide; Cb: carboplatin; H: trastuzumab; P: pertuzumab; 21 days is a cycle.

**Supplementary Table 2.** NACT protocol for external validation patients.

| NACT regimen | AC-T | AC | TAC | TCbHP | THP | AC-TH | TCbH | AC-THP | | TH |
| --- | --- | --- | --- | --- | --- | --- | --- | --- | --- | --- |
| N (Total=129) | 46 | 34 | 25 | 1 | 3 | 5 | 2 | 5 | 8 | |
| Patients with protocol change (n=6) | | AC (one cycle) changed to TAC (there cycles)  AC (one cycle) changed to TAC (four cycles)  AC (two cycles) changed to TAC (there cycles)  TAC (four cycles) changed to TCb (two cycles)  AC (four cycles)-T (there cycles) changed to TH (one cycle)  TCbH (four cycles) changed to TCbHP (two cycles) | | | | | | | | |
| Note: 16 patients with HER2 (+) did not receive targeted therapy (H or HP) | | | | | | | | | | |

Abbreviations: T: taxane; A: anthracycline; C: cyclophosphamide; Cb: carboplatin; H: trastuzumab; P: pertuzumab; 21 days is a cycle.

**Supplementary Table 3.** Relationship between MetS and clinical characteristics under different menstrual States.

|  | Non-MetS (% ) | MetS (% ) | Total | P value |
| --- | --- | --- | --- | --- |
| Postmenopausa | 45 (60.81%) | 29 (39.19%) | 74 |  |
| Tumor size |  |  |  | 0.088 |
| T≤2 cm | 7 | 3 | 10 |  |
| 2 cm＜T≤5 cm | 36 | 21 | 57 |  |
| T＞5 cm | 2 | 5 | 7 |  |
| Lymph node |  | l |  | 0.486 |
| Negative | 17 | 10 | 27 |  |
| Positive | 28 | 19 | 47 |  |
| Clinical stage |  |  |  | 0.175 |
| I | 2 | 0 | 2 |  |
| ⅡA | 19 | 12 | 31 |  |
| ⅡB | 22 | 13 | 35 |  |
| Ⅲ | 2 | 4 | 6 |  |
| Premenopaus | 59 (77.63%) | 17 (22.37%) | 76 |  |
| Tumor size |  |  |  | 0.339 |
| T≤2 cm | 12 | 2 | 14 |  |
| 2 cm＜T≤5 cm | 42 | 14 | 56 |  |
| T＞5 cm | 5 | 1 | 6 |  |
| Lymph node |  |  |  | 0.131 |
| Negative | 17 | 2 | 19 |  |
| Positive | 42 | 15 | 57 |  |
| Clinical stage |  |  |  | 0.091 |
| I | 2 | 0 | 2 |  |
| ⅡA | 25 | 4 | 29 |  |
| ⅡB | 27 | 12 | 39 |  |
| Ⅲ | 5 | 1 | 6 |  |

**Supplementary Table 4.** Univariate and multivariate analysis of laboratory and clinical indicators with BCNACT clinical response.

| Indicators | Total | clinical response | | Univariate analysis | Multivariate analysis (Hosmer Lemeshow test, P=0.563) | |
| --- | --- | --- | --- | --- | --- | --- |
|  |  | Poor | Good | P value | RR (95% CI) | P value |
|  | 150 | 33 | 117 |  |  |  |
| Age, y |  |  |  | 0.102 |  | **0.035** |
| ＜50 | 65 | 18 | 47 |  | 1 (Reference) |  |
| ≥50 | 85 | 15 | 70 |  | 0.208 (0.048-0.895 |  |
| BMI, kg/m^2^ |  |  |  | **0.007** |  | **0.015** |
| ＜25 | 94 | 27 | 67 |  | 1 (Reference) |  |
| 25≤BMI＜30 | 49 | 4 | 45 |  | 0.117 (0.026-0.537) | **0.006** |
| ≥30 | 7 | 2 | 5 |  | 0.134 (0.011-1.594) | 0.112 |
| Menopausal status |  |  |  | 0.465 |  | 0.262 |
| Premenopausal | 76 | 16 | 60 |  | 1 (Reference) |  |
| Postmenopausal | 74 | 17 | 57 |  | 2.343 (0.530-10.360) |  |
| WC, cm |  |  |  | 0.387 |  | 0.801 |
| ≤78.25 | 51 | 10 | 41 |  | 1 (Reference) |  |
| ＞78.25 | 99 | 23 | 76 |  | 0.837 (0.210-3.334) |  |
| FBG, mmol/L |  |  |  | **0.020** |  | 0.133 |
| ≤5.415 | 102 | 17 | 85 |  | 1 (Reference) |  |
| ＞5.415 | 48 | 16 | 32 |  | 2.546 (0.753-8.606) |  |
| Blood pressure |  |  |  |  |  |  |
| SBP, mmHg |  |  |  | 0.177 |  | 0.187 |
| ≤118.50 | 34 | 5 | 29 |  | 1 (Reference) |  |
| ＞118.50 | 116 | 28 | 88 |  | 2.539 (0.635-10.146) |  |
| DBP, mmHg |  |  |  | 0.180 |  | 0.100 |
| ≤100.5 | 143 | 30 | 113 |  | 1 (Reference) |  |
| ＞100.5 | 7 | 3 | 4 |  | 4.629 (0.745-28.769) |  |
| Lipid profile |  |  |  |  |  |  |
| TG, mmol/L |  |  |  | **0.044** |  | **0.036** |
| ≤1.3 | 81 | 13 | 68 |  | 1 (Reference) |  |
| ＞1.3 | 69 | 20 | 49 |  | 3.349 (1.080-10.385) |  |
| HDL-C, mmol/L |  |  |  | 0.233 |  | 0.292 |
| ＜0.955 | 22 | 3 | 19 |  | 1 (Reference) |  |
| ≥0.955 | 128 | 30 | 98 |  | 0.385 (0.065-2.274) |  |
| TC, mmol/L |  |  |  | 0.312 |  |  |
| ≤5.905 | 137 | 29 | 108 |  |  |  |
| ＞5.905 | 13 | 4 | 9 |  |  |  |
| LDL-C, mmol/L |  |  |  | 0.201 |  |  |
| ≤4.18 | 139 | 29 | 110 |  |  |  |
| ＞4.18 | 11 | 4 | 7 |  |  |  |
| UA, μmol/L |  |  |  | **0.008** |  | 0.126 |
| ≤408.5 | 145 | 29 | 116 |  | 1 (Reference) |  |
| ＞408.5 | 5 | 4 | 1 |  | 9.592 (0.530-173.565) |  |
| Cr, μmol/L |  |  |  | 0.522 |  |  |
| ≤60.9 | 97 | 21 | 76 |  |  |  |
| ＞60.9 | 53 | 12 | 41 |  |  |  |
| LDH, U/L |  |  |  | **0.049** |  | 0.097 |
| ≤226 | 133 | 26 | 107 |  | 1 (Reference) |  |
| ＞226 | 17 | 7 | 10 |  | 3.754 (0.786-17.936) |  |
| Inflammation |  |  |  |  |  |  |
| NLR |  |  |  | 0.308 |  |  |
| ≤2.085 | 58 | 11 | 47 |  |  |  |
| ＞2.085 | 92 | 22 | 70 |  |  |  |
| LMR |  |  |  | 0.395 |  |  |
| ≤5.525 | 69 | 14 | 55 |  |  |  |
| ＞5.525 | 81 | 19 | 62 |  |  |  |
| PLR |  |  |  | 0.082 |  |  |
| ≤119.655 | 49 | 7 | 42 |  |  |  |
| ＞119.655 | 101 | 26 | 75 |  |  |  |
| Tumor size |  |  |  | 0.354 |  |  |
| T≤2 cm | 24 | 6 | 18 |  |  |  |
| 2 cm＜T≤5 cm | 113 | 22 | 91 |  |  |  |
| T＞5 cm | 13 | 5 | 8 |  |  |  |
| Lymph node |  |  |  | 0.401 |  |  |
| Negative | 46 | 9 | 37 |  |  |  |
| Positive | 104 | 24 | 80 |  |  |  |
| Clinical stage |  |  |  | 0.268 |  |  |
| I | 4 | 1 | 3 |  |  |  |
| ⅡA | 60 | 12 | 48 |  |  |  |
| ⅡB | 74 | 16 | 58 |  |  |  |
| Ⅲ | 12 | 4 | 8 |  |  |  |
| Pathogenic type |  |  |  | 0.271 |  |  |
| Invasive carcinoma | 102 | 19 | 83 |  |  |  |
| Invasive carcinoma with ductal carcinoma | 38 | 12 | 26 |  |  |  |
| Others | 10 | 2 | 8 |  |  |  |
| Molecular subtype |  |  |  | **0.005** |  |  |
| HER2+/HR+ | 29 | 5 | 24 |  |  |  |
| HER2+/HR- | 39 | 2 | 37 |  |  |  |
| Luminal | 67 | 21 | 46 |  |  |  |
| TNBC | 15 | 5 | 10 |  |  |  |
| ER |  |  |  | 0.092 |  |  |
| Negative | 58 | 9 | 49 |  |  |  |
| Positive | 92 | 24 | 68 |  |  |  |
| PR |  |  |  | 0.347 |  |  |
| Negative | 84 | 17 | 67 |  |  |  |
| Positive | 66 | 16 | 50 |  |  |  |
| HER-2 |  |  |  | **＜0.001** |  | **＜0.001** |
| Negative | 82 | 27 | 55 |  | 1 (Reference) |  |
| Positive | 68 | 6 | 62 |  | 0.082 (0.021-0.318) |  |
| Ki-67 |  |  |  | 0.605 |  |  |
| Negative | 6 | 1 | 5 |  |  |  |
| Positive | 144 | 32 | 112 |  |  |  |
| TNBC |  |  |  | 0.210 |  |  |
| Yes | 15 | 5 | 10 |  |  |  |
| No | 135 | 28 | 107 |  |  |  |
| NACT regimen |  |  |  | 0.108 |  |  |
| Nontaxane based | 9 | 4 | 5 |  |  |  |
| Taxane based | 141 | 29 | 112 |  |  |  |
| MetS |  |  |  | **0.033** |  | 0.217 |
| No | 104 | 18 | 86 |  | 1 (Reference) |  |
| Yes | 46 | 15 | 31 |  | 2.537 (0.580-11.103) |  |

Abbreviations: RR, risk ratio; CI, confidence interval; BMI, body mass index; WC, waist circumference; FBG, fasting blood glucose; SBP, systolic blood pressure; DBP, diastolic blood pressure; TG, triglycerides; HDL-C, high-density lipoprotein cholesterol; TC, total cholesterol; LDL-C; low-density lipoprotein cholesterol; UA, uric acid; Cr, creatinine; LDH, lactate dehydrogenase; NLR, neutrophil-lymphocyte ratio; LMR, lymphocyte-monocyte ratio; PLR, platelet-lymphocyte ratio; HER2, human epidermal growth factor 2; HR, hormone receptor; TNBC, triple negative breast cancer; ER, estrogen receptor; PR, progesterone receptor; NACT, neoadjuvant chemotherapy.

**Supplementary table 5.** Univariate and multivariate analysis of laboratory and clinical indicators with BCNACT pathologic response.

| Indicators | Total | pathologic response | | Univariate analysis | Multivariate analysis (Hosmer Lemeshow test, P=0.854) | |
| --- | --- | --- | --- | --- | --- | --- |
|  |  | Poor | Good | P value | RR (95% CI) | P value |
|  | 150 | 67 | 83 |  |  |  |
| Mean age, y |  |  |  | 0.207 |  | 0.226 |
| ＜50 | 65 | 32 | 33 |  | 1 (Reference) |  |
| ≥50 | 85 | 35 | 50 |  | 0.362 (0.070-1.875) |  |
| BMI, kg/m^2^ |  |  |  | 0.097 |  | 0.073 |
| ＜25 | 94 | 39 | 55 |  | 1 (Reference) |  |
| 25≤BMI＜30 | 49 | 22 | 27 |  | 0.207 (0.052-1.016) | 0.051 |
| ≥30 | 7 | 6 | 1 |  | 1.108 (0.015-80.834) | 0.963 |
| Menopausal status |  |  |  | 0.428 |  | 0.572 |
| Premenopausal | 76 | 35 | 41 |  | 1 (Reference) |  |
| Postmenopausal | 74 | 32 | 42 |  | 0.623 (0.121-3.217) |  |
| WC, cm |  |  |  | **＜0.001** |  | 0.059 |
| ≤80.4 | 66 | 12 | 54 |  | 1 (Reference) |  |
| ＞80.4 | 84 | 55 | 29 |  | 4.780 (0.908-43.117) |  |
| FBG, mmol/L |  |  |  | 0.070 |  | 0.535 |
| ≤5.73 | 121 | 50 | 71 |  | 1 (Reference) |  |
| ＞5.73 | 29 | 17 | 12 |  | 1.656 (0.336-8.164) |  |
| Blood pressure |  |  |  |  |  |  |
| SBP, mmHg |  |  |  | 0.124 |  | 0.141 |
| ≤119.50 | 37 | 13 | 24 |  | 1 (Reference) |  |
| ＞119.50 | 113 | 54 | 59 |  | 2.835 (0.707-11.369) |  |
| DBP, mmHg |  |  |  | 0.354 |  | 0.878 |
| ≤97.50 | 139 | 61 | 78 |  | 1 (Reference) |  |
| ＞97.50 | 11 | 6 | 5 |  | 1.192 (0.126-11.276) |  |
| Lipid profile |  |  |  |  |  |  |
| TG, mmol/L |  |  |  | **0.012** |  | **0.007** |
| ≤0.865 | 34 | 9 | 25 |  | 1 (Reference) |  |
| ＞0.865 | 116 | 58 | 58 |  | 8.863 (1.794-43.779) |  |
| HDL-C, mmol/L |  |  |  | 0.382 |  | 0.623 |
| ＜0.385 | 143 | 63 | 80 |  | 1 (Reference) |  |
| ≥0.385 | 7 | 4 | 3 |  | 3.963 (0.016-962.918) |  |
| TC, mmol/L |  |  |  | 0.420 |  |  |
| ≤6.75 | 147 | 65 | 82 |  |  |  |
| ＞6.75 | 3 | 2 | 1 |  |  |  |
| LDL-C, mmol/L |  |  |  | 0.553 |  |  |
| ≤1.435 | 1 | 0 | 1 |  |  |  |
| ＞1.435 | 149 | 67 | 82 |  |  |  |
| UA, μmol/L |  |  |  | 0.060 |  | 0.431 |
| ≤179.5 | 8 | 1 | 7 |  | 1 (Reference) |  |
| ＞179.5 | 142 | 66 | 76 |  | 64.517 (0.002-2.037*10^6^) |  |
| Cr, μmol/L |  |  |  | **0.029** |  | 0.118 |
| ≤64.95 | 121 | 49 | 72 |  | 1 (Reference) |  |
| ＞64.95 | 29 | 18 | 11 |  | 3.203 (0.745-13.770) |  |
| LDH, U/L |  |  |  | 0.225 |  |  |
| ≤136.50 | 13 | 4 | 9 |  |  |  |
| ＞136.50 | 137 | 63 | 74 |  |  |  |
| Inflammation |  |  |  |  |  |  |
| NLR |  |  |  | **0.007** |  | 0.999 |
| ≤8.5 | 144 | 61 | 83 |  | 1 (Reference) |  |
| ＞8.5 | 6 | 6 | 0 |  | 3.597*10^9^ (0.000- ~) |  |
| LMR |  |  |  | 0.098 |  |  |
| ≤9.765 | 138 | 59 | 79 |  |  |  |
| ＞9.765 | 12 | 8 | 4 |  |  |  |
| PLR |  |  |  | 0.198 |  |  |
| ≤453.96 | 148 | 65 | 83 |  |  |  |
| ＞453.96 | 2 | 2 | 0 |  |  |  |
| Tumor size |  |  |  | 0.067 |  |  |
| T≤2 cm | 24 | 9 | 15 |  |  |  |
| 2 cm＜T≤5 cm | 113 | 49 | 64 |  |  |  |
| T＞5 cm | 13 | 9 | 4 |  |  |  |
| Lymph node |  |  |  | 0.494 |  |  |
| Negative | 46 | 20 | 26 |  |  |  |
| Positive | 104 | 47 | 57 |  |  |  |
| Clinical stage |  |  |  | 0.134 |  |  |
| I | 4 | 1 | 3 |  |  |  |
| ⅡA | 60 | 25 | 35 |  |  |  |
| ⅡB | 74 | 34 | 40 |  |  |  |
| Ⅲ | 12 | 7 | 5 |  |  |  |
| Pathogenic type |  |  |  | **0.038** |  | 0.602 |
| Invasive carcinoma | 102 | 46 | 56 |  | 1 (Reference) |  |
| Invasive carcinoma with ductal carcinoma | 38 | 13 | 25 |  | 1.441 (0.375-5.541) | 0.595 |
| Others | 10 | 8 | 2 |  | 9.758 (0.069-1376.146) | 0.340 |
| Molecular subtype |  |  |  | **＜0.001** |  |  |
| HER2+/HR+ | 29 | 10 | 19 |  |  |  |
| HER2+/HR- | 39 | 5 | 34 |  |  |  |
| Luminal | 67 | 44 | 23 |  |  |  |
| TNBC | 15 | 8 | 7 |  |  |  |
| ER |  |  |  | **0.006** |  | 0.300 |
| Negative | 58 | 18 | 40 |  | 1 (Reference) |  |
| Positive | 92 | 49 | 43 |  | 0.443 (0.095-2.066) |  |
| PR |  |  |  | **0.001** |  | **0.046** |
| Negative | 84 | 28 | 56 |  | 1 (Reference) |  |
| Positive | 66 | 39 | 27 |  | 4.236 (1.024-17.524) |  |
| HER-2 |  |  |  | **＜0.001** |  | **＜0.001** |
| Negative | 82 | 53 | 29 |  | 1 (Reference) |  |
| Positive | 68 | 14 | 54 |  | 0.072 (0.018-0.291) |  |
| Ki-67 |  |  |  | 0.553 |  |  |
| Negative | 6 | 3 | 3 |  |  |  |
| Positive | 144 | 64 | 80 |  |  |  |
| TNBC |  |  |  | 0.329 |  |  |
| Yes | 15 | 8 | 7 |  |  |  |
| No | 135 | 59 | 76 |  |  |  |
| NACT regimen |  |  |  | **0.043** |  | 0.392 |
| Nontaxane based | 9 | 7 | 2 |  | 1 (Reference) |  |
| Taxane based | 141 | 60 | 81 |  | 0.361 (0.035-3.722) |  |
| MetS |  |  |  | **＜0.001** |  | 0.458 |
| No | 104 | 33 | 71 |  | 1 (Reference) |  |
| Yes | 46 | 34 | 12 |  | 1.782 (0.388-8.183) |  |

Abbreviations: RR, risk ratio; CI, confidence interval; BMI, body mass index; WC, waist circumference; FBG, fasting blood glucose; SBP, systolic blood pressure; DBP, diastolic blood pressure; TG, triglycerides; HDL-C, high-density lipoprotein cholesterol; TC, total cholesterol; LDL-C; low-density lipoprotein cholesterol; UA, uric acid; Cr, creatinine; LDH, lactate dehydrogenase; NLR, neutrophil-lymphocyte ratio; LMR, lymphocyte-monocyte ratio; PLR, platelet-lymphocyte ratio; HER2, human epidermal growth factor 2; HR, hormone receptor; TNBC, triple negative breast cancer; ER, estrogen receptor; PR, progesterone receptor; NACT, neoadjuvant chemotherapy.

**Supplementary Table 6.** Relationship between fast blood glucose, serum lipid and the efficacy of BCNACT in different ER states.

|  | pCR | | | clinical response | | | pathologic response | | |
| --- | --- | --- | --- | --- | --- | --- | --- | --- | --- |
|  | No | Yes | P value | Poor | Good | P value | Poor | Good | P value |
| Total (ER+) | 71 | 21 |  | 24 | 68 |  | 49 | 43 |  |
| FBG, mmol/L | | | 0.081 |  |  | **0.027** |  |  | 0.407 |
| low | 54 | 12 |  | 13 | 53 |  | 39 | 36 |  |
| high | 17 | 9 |  | 11 | 15 |  | 10 | 7 |  |
| Lipid profile, mmol/L | | |  |  |  |  |  |  |  |
| TG, |  |  | 0.103 |  |  | 0.090 |  |  | **0.015** |
| low | 50 | 11 |  | 10 | 41 |  | 5 | 13 |  |
| high | 21 | 10 |  | 14 | 27 |  | 44 | 30 |  |
| HDL-C |  |  | 0.138 |  |  | 0.146 |  |  | 0.359 |
| low | 63 | 16 |  | 2 | 14 |  | 3 | 1 |  |
| high | 8 | 5 |  | 22 | 54 |  | 46 | 42 |  |
| TC |  |  | **0.021** |  |  | 0.431 |  |  | 0.281 |
| low | 50 | 9 |  | 21 | 62 |  | 47 | 43 |  |
| high | 21 | 12 |  | 3 | 6 |  | 2 | 0 |  |
| LDL-C |  |  | **0.028** |  |  | 0.261 |  |  | 0.467 |
| low | 49 | 9 |  | 21 | 64 |  | 0 | 1 |  |
| high | 22 | 12 |  | 3 | 4 |  | 49 | 42 |  |
| Total (ER-) | 32 | 26 |  | 9 | 49 |  | 18 | 40 |  |
| FBG, mmol/L | | | 0.577 |  |  | 0.207 |  |  | **0.029** |
| low | 20 | 16 |  | 4 | 32 |  | 11 | 35 |  |
| high | 12 | 10 |  | 5 | 17 |  | 7 | 5 |  |
| Lipid profile, mmol/L | | |  |  |  |  |  |  |  |
| TG, |  |  | 0.423 |  |  | 0.201 |  |  | 0.391 |
| low | 19 | 17 |  | 3 | 27 |  | 4 | 12 |  |
| high | 13 | 9 |  | 6 | 22 |  | 14 | 28 |  |
| HDL-C |  |  | 0.216 |  |  | 0.655 |  |  | 0.680 |
| low | 25 | 17 |  | 1 | 5 |  | 17 | 38 |  |
| high | 7 | 9 |  | 8 | 44 |  | 1 | 2 |  |
| TC |  |  | 0.417 |  |  | 0.501 |  |  | 0.690 |
| low | 18 | 13 |  | 8 | 46 |  | 18 | 39 |  |
| high | 14 | 13 |  | 1 | 3 |  | 0 | 1 |  |
| LDL-C |  |  | 0.459 |  |  | 0.501 |  |  | - |
| low | 20 | 15 |  | 8 | 46 |  | 0 | 0 |  |
| high | 12 | 11 |  | 1 | 3 |  | 18 | 40 |  |

Abbreviations: pCR, pathologic complete response; ER, estrogen receptor; FBG, fasting blood glucose; TG, triglycerides; HDL-C, high-density lipoprotein cholesterol; TC, total cholesterol; LDL-C; low-density lipoprotein cholesterol.

**Supplementary Table 7.** Population and clinicopathologic characteristics of external validation patients.

| Characteristics | Non-MetS (% / ±SD) | MetS (% / ±SD) | Total | P value |
| --- | --- | --- | --- | --- |
|  | 80 (59.26%） | 55(40.74%） | 135 |  |
| Mean age, y |  |  |  |  |
| ＜50 | 45 | 19 | 64 | **0.010** |
| ≥50 | 35 | 36 | 71 |  |
| BMI, kg/m^2^ |  |  |  |  |
| ＜25 | 58 | 25 | 83 | **0.001** |
| 25＜T≤30 | 19 | 25 | 44 |  |
| T＞30 | 3 | 5 | 8 |  |
| Menopausal status |  |  |  |  |
| Premenopausal | 52 | 26 | 78 | **0.031** |
| Postmenopausal | 28 | 29 | 57 |  |
| WC, cm |  |  |  |  |
| ≤80 | 48 | 0 | 48 | **＜0.001** |
| ＞80 | 32 | 55 | 87 |  |
| FBG, mmol/L |  |  |  |  |
| ≤5.6 | 71 | 26 | 97 | **＜0.001** |
| ＞5.6 | 9 | 29 | 38 |  |
| Blood pressure |  |  |  |  |
| SBP, mmHg |  |  |  |  |
| ≤130 | 59 | 15 | 74 | **＜0.001** |
| ＞13 | 21 | 40 | 61 |  |
| DBP, mmHg |  |  |  |  |
| ≤85 | 72 | 34 | 106 | **＜0.001** |
| ＞85 | 8 | 21 | 29 |  |
| Lipid profile |  |  |  |  |
| TG, mmol/L |  |  |  |  |
| ≤1.7 | 72 | 30 | 102 | **＜0.001** |
| ＞1.7 | 8 | 25 | 33 |  |
| HDL-C, mmol/L |  |  |  |  |
| ＜1.3 | 34 | 16 | 50 | 0.080 |
| ≥ 1.3 | 46 | 39 | 85 |  |
| TC, mmol/L | 4.39±0.77 | 4.91±0.92 |  | **＜0.001** |
| LDL-C, mmol/L | 2.78±0.56 | 3.27±0.69 |  | **＜0.001** |
| UA, μmol/L | 248.26±54.06 | 272.05±69.24 |  | **0.027** |
| Cr, μmol/L | 60.64±10.45 | 61.16±7.85 |  | 0.755 |
| LDH, U/L | 175.50±38.80 | 192.87±40.07 |  | **0.013** |
| Inflammation |  |  |  |  |
| NLR | 3.45±2.74 | 2.74±2.00 |  | 0.103 |
| LMR | 8.92±12.36 | 7.18±13.23 |  | 0.436 |
| PLR | 166.90±57.75 | 150.30±70.06 |  | 0.135 |
| Tumor size |  |  |  | 0.289 |
| T≤2 cm | 14 | 11 | 25 |  |
| 2 cm＜T≤5 cm | 59 | 41 | 100 |  |
| T＞5 cm | 7 | 3 | 10 |  |
| Lymph node |  |  |  | 0.273 |
| Negative | 33 | 19 | 52 |  |
| Positive | 47 | 36 | 83 |  |
| Clinical stage |  |  |  | 0.431 |
| I | 8 | 2 | 10 |  |
| ⅡA | 30 | 27 | 57 |  |
| ⅡB | 34 | 21 | 55 |  |
| Ⅲ | 8 | 5 | 13 |  |
| Pathogenic type |  |  |  | 1.000 |
| Invasive carcinoma | 74 | 51 | 125 |  |
| Invasive carcinoma with ductal carcinoma | 4 | 2 | 6 |  |
| Others | 2 | 2 | 4 |  |
| Molecular subtype |  |  |  | 0.354 |
| HER2+/HR+ | 19 | 8 | 27 |  |
| HER2+/HR- | 10 | 4 | 14 |  |
| Luminal | 39 | 34 | 73 |  |
| TNBC | 12 | 9 | 21 |  |
| ER |  |  |  | 0.405 |
| Negative | 22 | 17 | 39 |  |
| Positive | 58 | 38 | 96 |  |
| PR |  |  |  | 0.348 |
| Negative | 33 | 20 | 53 |  |
| Positive | 47 | 35 | 82 |  |
| HER-2 |  |  |  | 0.054 |
| Negative | 51 | 43 | 94 |  |
| Positive | 29 | 12 | 41 |  |
| Ki-67 |  |  |  | 0.459 |
| Negative | 9 | 5 | 14 |  |
| Positive | 71 | 50 | 121 |  |
| TNBC |  |  |  | 0.506 |
| Yes | 12 | 9 | 21 |  |
| No | 68 | 46 | 114 |  |
| NACT regimen |  |  |  | 0.143 |
| Nontaxane based | 17 | 17 | 34 |  |
| Taxane based | 63 | 38 | 101 |  |

Abbreviations: MetS, metabolic syndrome; SD, standard deviation; BMI, body mass index; WC, waist circumference; FBG, fasting blood glucose; SBP, systolic blood pressure; DBP, diastolic blood pressure; TG, triglycerides; HDL-C, high-density lipoprotein cholesterol; TC, total cholesterol; LDL-C; low-density lipoprotein cholesterol; UA, uric acid; Cr, creatinine; LDH, lactate dehydrogenase; NLR, neutrophil-lymphocyte ratio; LMR, lymphocyte-monocyte ratio; PLR, platelet-lymphocyte ratio; HER2, human epidermal growth factor 2; HR, hormone receptor; TNBC, triple negative breast cancer; ER, estrogen receptor; PR, progesterone receptor; NACT, neoadjuvant chemotherapy.

**Supplementary Table 8.** Relationship between MetS and clinical characteristics of external validation patients under different menstrual States.

|  | Non-MetS (% ) | MetS (% ) | Total | P value |
| --- | --- | --- | --- | --- |
| Postmenopausa | 28 (49.12%) | 29 (50.88%) | 57 |  |
| Tumor size |  |  |  | 0.486 |
| T≤2 cm | 5 | 4 | 9 |  |
| 2 cm＜T≤5 cm | 20 | 23 | 43 |  |
| T＞5 cm | 3 | 2 | 5 |  |
| Lymph node |  | l |  | 0.543 |
| Negative | 11 | 12 | 23 |  |
| Positive | 17 | 17 | 34 |  |
| Clinical stage |  |  |  | 0.385 |
| I | 4 | 2 | 6 |  |
| ⅡA | 7 | 13 | 20 |  |
| ⅡB | 14 | 10 | 24 |  |
| Ⅲ | 3 | 4 | 7 |  |
| Premenopaus | 52 (66.67%) | 26 (33.33%) | 78 |  |
| Tumor size |  |  |  | 0.162 |
| T≤2 cm | 9 | 7 | 16 |  |
| 2 cm＜T≤5 cm | 39 | 18 | 57 |  |
| T＞5 cm | 4 | 1 | 5 |  |
| Lymph node |  |  |  | 0.141 |
| Negative | 22 | 7 | 29 |  |
| Positive | 30 | 19 | 49 |  |
| Clinical stage |  |  |  | 0.507 |
| I | 4 | 0 | 4 |  |
| ⅡA | 23 | 14 | 37 |  |
| ⅡB | 20 | 11 | 31 |  |
| Ⅲ | 5 | 1 | 6 |  |

**Supplementary Table 9.** Univariate and multivariate analysis of laboratory and clinical indicators with BCNACT clinical response on external validation patients.

| Indicators | Total | clinical response | | Univariate analysis | Multivariate analysis (Hosmer Lemeshow test, P=0.193) | |
| --- | --- | --- | --- | --- | --- | --- |
|  |  | Poor | Good | P value | RR (95% CI) | P value |
|  | 135 | 59 | 76 |  |  |  |
| Age, y |  |  |  | **0.012** |  | 0.343 |
| ＜50 | 64 | 21 | 43 |  | 1 (Reference) |  |
| ≥50 | 71 | 38 | 33 |  | 1.833 (0.524-6.409) |  |
| BMI, kg/m^2^ |  |  |  | **0.014** |  | 0.394 |
| ＜25 | 83 | 30 | 53 |  | 1 (Reference) |  |
| 25≤BMI＜30 | 44 | 24 | 20 |  | 2.013 (0.708-5.725) | 0.190 |
| ≥30 | 8 | 5 | 3 |  | 0.894 (0.099-8.112) | 0.921 |
| Menopausal status |  |  |  | **0.010** |  | 0.089 |
| Premenopausal | 78 | 27 | 51 |  | 1 (Reference) |  |
| Postmenopausal | 57 | 32 | 25 |  | 2.888 (0.850-9.814) |  |
| WC, cm |  |  |  | 0.273 |  | 0.859 |
| ≤78.25 | 32 | 12 | 20 |  | 1 (Reference) |  |
| ＞78.25 | 103 | 47 | 56 |  | 0.894 (0.260-3.077) |  |
| FBG, mmol/L |  |  |  | 0.113 |  | 0.709 |
| ≤5.415 | 91 | 36 | 55 |  | 1 (Reference) |  |
| ＞5.415 | 44 | 23 | 21 |  | 0.813 (0.274-2.414) |  |
| Blood pressure |  |  |  |  |  |  |
| SBP, mmHg |  |  |  | **0.020** |  | 0.433 |
| ≤118.50 | 28 | 7 | 21 |  | 1 (Reference) |  |
| ＞118.50 | 107 | 52 | 55 |  | 1.670 (0.463-6.024) |  |
| DBP, mmHg |  |  |  | 0.381 |  | 0.327 |
| ≤100.5 | 130 | 56 | 74 |  | 1 (Reference) |  |
| ＞100.5 | 5 | 3 | 2 |  | 3.965 (0.252-62.318) |  |
| Lipid profile |  |  |  |  |  |  |
| TG, mmol/L |  |  |  | 0.151 |  | 0.194 |
| ≤1.3 | 72 | 28 | 44 |  | 1 (Reference) |  |
| ＞1.3 | 63 | 31 | 32 |  | 0.480 (0.159-1.453) |  |
| HDL-C, mmol/L |  |  |  | 0.387 |  | 0.169 |
| ＜0.955 | 126 | 56 | 70 |  | 1 (Reference) |  |
| ≥0.955 | 9 | 3 | 6 |  | 0.240 (0.031-1.832) |  |
| TC, mmol/L |  |  |  | **0.019** |  | 0.626 |
| ≤5.905 | 125 | 51 | 74 |  | 1 (Reference) |  |
| ＞5.905 | 10 | 8 | 2 |  | 1.693 (0.203-14.094) |  |
| LDL-C, mmol/L |  |  |  | **0.034** |  | 0.999 |
| ≤4.18 | 131 | 55 | 76 |  | 1 (Reference) |  |
| ＞4.18 | 4 | 4 | 0 |  | 1.841*10^8^ (0.000- ~) |  |
| UA, μmol/L |  |  |  | 0.685 |  | 0.304 |
| ≤408.5 | 133 | 58 | 75 |  | 1 (Reference) |  |
| ＞408.5 | 2 | 1 | 1 |  | 0.114 (0.002-7.129) |  |
| Cr, μmol/L |  |  |  | 0.167 |  |  |
| ≤60.9 | 68 | 33 | 35 |  |  |  |
| ＞60.9 | 67 | 26 | 41 |  |  |  |
| LDH, U/L |  |  |  | 0.179 |  |  |
| ≤226 | 121 | 55 | 66 |  |  |  |
| ＞226 | 14 | 4 | 10 |  |  |  |
| Inflammation |  |  |  |  |  |  |
| NLR |  |  |  | 0.363 |  |  |
| ≤2.085 | 47 | 22 | 25 |  |  |  |
| ＞2.085 | 88 | 37 | 51 |  |  |  |
| LMR |  |  |  | 0.539 |  |  |
| ≤5.525 | 75 | 33 | 42 |  |  |  |
| ＞5.525 | 60 | 26 | 34 |  |  |  |
| PLR |  |  |  | 0.363 |  |  |
| ≤119.655 | 47 | 22 | 25 |  |  |  |
| ＞119.655 | 88 | 37 | 51 |  |  |  |
| Tumor size |  |  |  | 0.135 |  |  |
| T≤2 cm | 25 | 13 | 12 |  |  |  |
| 2 cm＜T≤5 cm | 100 | 43 | 57 |  |  |  |
| T＞5 cm | 10 | 3 | 7 |  |  |  |
| Lymph node |  |  |  | 0.332 |  |  |
| Negative | 52 | 21 | 31 |  |  |  |
| Positive | 83 | 38 | 45 |  |  |  |
| Clinical stage |  |  |  | 0.422 |  |  |
| I | 10 | 5 | 5 |  |  |  |
| ⅡA | 57 | 24 | 33 |  |  |  |
| ⅡB | 55 | 23 | 32 |  |  |  |
| Ⅲ | 13 | 7 | 6 |  |  |  |
| Pathogenic type |  |  |  | 0.684 |  |  |
| Invasive carcinoma | 125 | 56 | 69 |  |  |  |
| Invasive carcinoma with ductal carcinoma | 6 | 2 | 4 |  |  |  |
| Others | 4 | 1 | 3 |  |  |  |
| Molecular subtype |  |  |  | **0.003** |  |  |
| HER2+/HR+ | 27 | 6 | 21 |  |  |  |
| HER2+/HR- | 14 | 3 | 11 |  |  |  |
| Luminal | 73 | 42 | 31 |  |  |  |
| TNBC | 21 | 8 | 13 |  |  |  |
| ER |  |  |  | 0.087 |  |  |
| Negative | 39 | 13 | 26 |  |  |  |
| Positive | 96 | 46 | 50 |  |  |  |
| PR |  |  |  | **0.048** |  | 0.173 |
| Negative | 53 | 18 | 35 |  | 1 (Reference) |  |
| Positive | 82 | 41 | 41 |  | 1.981 (0.741-5.295) |  |
| HER-2 |  |  |  | **0.001** |  | **0.004** |
| Negative | 94 | 50 | 44 |  | 1 (Reference) |  |
| Positive | 41 | 9 | 32 |  | 0.192 (0.063-0.585) |  |
| Ki-67 |  |  |  | 0.589 |  |  |
| Negative | 14 | 6 | 8 |  |  |  |
| Positive | 121 | 53 | 68 |  |  |  |
| TNBC |  |  |  | 0.212 |  |  |
| Yes | 21 | 7 | 14 |  |  |  |
| No | 114 | 52 | 62 |  |  |  |
| NACT regimen |  |  |  | **＜0.001** |  | **＜0.001** |
| Nontaxane based | 34 | 27 | 7 |  | 1 (Reference) |  |
| Taxane based | 101 | 32 | 69 |  | 0.060 (0.017-0.218) |  |
| MetS |  |  |  | **0.004** |  | 0.267 |
| No | 80 | 27 | 53 |  | 1 (Reference) |  |
| Yes | 55 | 32 | 23 |  | 1.996 (0.589-6.761) |  |

Abbreviations: RR, risk ratio; CI, confidence interval; BMI, body mass index; WC, waist circumference; FBG, fasting blood glucose; SBP, systolic blood pressure; DBP, diastolic blood pressure; TG, triglycerides; HDL-C, high-density lipoprotein cholesterol; TC, total cholesterol; LDL-C; low-density lipoprotein cholesterol; UA, uric acid; Cr, creatinine; LDH, lactate dehydrogenase; NLR, neutrophil-lymphocyte ratio; LMR, lymphocyte-monocyte ratio; PLR, platelet-lymphocyte ratio; HER2, human epidermal growth factor 2; HR, hormone receptor; TNBC, triple negative breast cancer; ER, estrogen receptor; PR, progesterone receptor; NACT, neoadjuvant chemotherapy.

**Supplementary Table 10.** Univariate and multivariate analysis of laboratory and clinical indicators with BCNACT pathologic response on external validation patients.

| Indicators | Total | pathologic response | | Univariate analysis | Multivariate analysis (Hosmer Lemeshow test, P=0.562) | |
| --- | --- | --- | --- | --- | --- | --- |
|  |  | Poor | Good | P value | RR (95% CI) | P value |
|  | 135 | 101 | 34 |  |  |  |
| Mean age, y |  |  |  | 0.439 |  | 0.502 |
| ＜50 | 64 | 47 | 17 |  | 1 (Reference) |  |
| ≥50 | 71 | 54 | 17 |  | 1.622 (0.395-6.661) |  |
| BMI, kg/m^2^ |  |  |  | 0.108 |  | 0.881 |
| ＜25 | 83 | 59 | 24 |  | 1 (Reference) |  |
| 25≤BMI＜30 | 44 | 35 | 9 |  | 0.825 (0.270-2.523) | 0.735 |
| ≥30 | 8 | 7 | 1 |  | 1.530 (0.126-18.511) | 0.738 |
| Menopausal status |  |  |  | 0.475 |  | 0.416 |
| Premenopausal | 78 | 59 | 19 |  | 1 (Reference) |  |
| Postmenopausal | 57 | 42 | 15 |  | 0.568 (0.145-2.223) |  |
| WC, cm |  |  |  | **0.004** |  | 0.052 |
| ≤80.4 | 48 | 29 | 19 |  | 1 (Reference) |  |
| ＞80.4 | 87 | 72 | 15 |  | 4.716 (0.974-17.796) |  |
| FBG, mmol/L |  |  |  | 0.094 |  | 0.507 |
| ≤5.73 | 102 | 73 | 29 |  | 1 (Reference) |  |
| ＞5.73 | 33 | 28 | 5 |  | 1.586 (0.406-6.196) |  |
| Blood pressure |  |  |  |  |  |  |
| SBP, mmHg |  |  |  | **0.039** |  | 0.129 |
| ≤119.50 | 34 | 21 | 13 |  | 1 (Reference) |  |
| ＞119.50 | 101 | 80 | 21 |  | 2.541 (0.763-8.461) |  |
| DBP, mmHg |  |  |  | 0.629 |  | 0.587 |
| ≤97.50 | 130 | 97 | 33 |  | 1 (Reference) |  |
| ＞97.50 | 5 | 4 | 1 |  | 2.397 (0.102-56.222) |  |
| Lipid profile |  |  |  |  |  |  |
| TG, mmol/L |  |  |  | 0.198 |  | 0.547 |
| ≤0.865 | 27 | 18 | 9 |  | 1 (Reference) |  |
| ＞0.865 | 108 | 83 | 25 |  | 1.456 (0.429-4.946) |  |
| HDL-C, mmol/L |  |  |  | - |  |  |
| ＜0.385 | 135 | 101 | 34 |  |  |  |
| ≥0.385 | 0 | 0 | 0 |  |  |  |
| TC, mmol/L |  |  |  | 0.748 |  |  |
| ≤6.75 | 134 | 100 | 34 |  |  |  |
| ＞6.75 | 1 | 1 | 0 |  |  |  |
| LDL-C, mmol/L |  |  |  | - |  |  |
| ≤1.435 | 0 | 0 | 0 |  |  |  |
| ＞1.435 | 135 | 101 | 34 |  |  |  |
| UA, μmol/L |  |  |  | 0.287 |  | 0.215 |
| ≤179.5 | 9 | 8 | 1 |  | 1 (Reference) |  |
| ＞179.5 | 126 | 93 | 33 |  | 0.221 (0.020-2.397) |  |
| Cr, μmol/L |  |  |  | 0.289 |  |  |
| ≤64.95 | 92 | 67 | 25 |  |  |  |
| ＞64.95 | 43 | 34 | 9 |  |  |  |
| LDH, U/L |  |  |  | 0.646 |  |  |
| ≤136.50 | 12 | 9 | 3 |  |  |  |
| ＞136.50 | 123 | 92 | 31 |  |  |  |
| Inflammation |  |  |  |  |  |  |
| NLR |  |  |  | 0.629 |  |  |
| ≤8.5 | 130 | 97 | 33 |  |  |  |
| ＞8.5 | 5 | 4 | 1 |  |  |  |
| LMR |  |  |  | 0.447 |  |  |
| ≤9.765 | 120 | 89 | 31 |  |  |  |
| ＞9.765 | 15 | 12 | 3 |  |  |  |
| PLR |  |  |  | - |  |  |
| ≤453.96 | 135 | 101 | 34 |  |  |  |
| ＞453.96 | 0 | 0 | 0 |  |  |  |
| Tumor size |  |  |  | 0.265 |  |  |
| T≤2 cm | 25 | 20 | 5 |  |  |  |
| 2 cm＜T≤5 cm | 100 | 74 | 26 |  |  |  |
| T＞5 cm | 10 | 7 | 3 |  |  |  |
| Lymph node |  |  |  | 0.282 |  |  |
| Negative | 52 | 37 | 15 |  |  |  |
| Positive | 83 | 64 | 19 |  |  |  |
| Clinical stage |  |  |  | 0.477 |  |  |
| I | 10 | 7 | 3 |  |  |  |
| ⅡA | 57 | 44 | 13 |  |  |  |
| ⅡB | 55 | 39 | 16 |  |  |  |
| Ⅲ | 13 | 11 | 2 |  |  |  |
| Pathogenic type |  |  |  | 0.716 |  |  |
| Invasive carcinoma | 125 | 92 | 33 |  |  |  |
| Invasive carcinoma with ductal carcinoma | 6 | 5 | 1 |  |  |  |
| Others | 4 | 4 | 0 |  |  |  |
| Molecular subtype |  |  |  | **＜0.001** |  |  |
| HER2+/HR+ | 27 | 16 | 11 |  |  |  |
| HER2+/HR- | 14 | 6 | 8 |  |  |  |
| Luminal | 73 | 66 | 7 |  |  |  |
| TNBC | 21 | 13 | 8 |  |  |  |
| ER |  |  |  | **0.008** |  | 0.870 |
| Negative | 39 | 23 | 16 |  | 1 (Reference) |  |
| Positive | 96 | 78 | 18 |  | 0.895 (0.239-3.357) |  |
| PR |  |  |  | **＜0.001** |  | **0.019** |
| Negative | 53 | 30 | 23 |  | 1 (Reference) |  |
| Positive | 82 | 71 | 11 |  | 4.831 (1.291-18.077) |  |
| HER-2 |  |  |  | **＜0.001** |  | **0.002** |
| Negative | 94 | 79 | 15 |  | 1 (Reference) |  |
| Positive | 41 | 22 | 19 |  | 0.198 (0.072-0.543) |  |
| Ki-67 |  |  |  | 0.489 |  |  |
| Negative | 14 | 10 | 4 |  |  |  |
| Positive | 121 | 91 | 30 |  |  |  |
| TNBC |  |  |  | 0.115 |  |  |
| Yes | 21 | 13 | 8 |  |  |  |
| No | 114 | 88 | 26 |  |  |  |
| NACT regimen |  |  |  | 0.077 |  |  |
| Nontaxane based | 34 | 29 | 5 |  |  |  |
| Taxane based | 101 | 72 | 29 |  |  |  |
| MetS |  |  |  | **0.014** |  | 0.483 |
| No | 80 | 54 | 26 |  | 1 (Reference) |  |
| Yes | 55 | 47 | 8 |  | 0.571 (0.119-2.734) |  |

Abbreviations: RR, risk ratio; CI, confidence interval; BMI, body mass index; WC, waist circumference; FBG, fasting blood glucose; SBP, systolic blood pressure; DBP, diastolic blood pressure; TG, triglycerides; HDL-C, high-density lipoprotein cholesterol; TC, total cholesterol; LDL-C; low-density lipoprotein cholesterol; UA, uric acid; Cr, creatinine; LDH, lactate dehydrogenase; NLR, neutrophil-lymphocyte ratio; LMR, lymphocyte-monocyte ratio; PLR, platelet-lymphocyte ratio; HER2, human epidermal growth factor 2; HR, hormone receptor; TNBC, triple negative breast cancer; ER, estrogen receptor; PR, progesterone receptor; NACT, neoadjuvant chemotherapy.

**Supplementary Table 11.** Relationship between fast blood glucose, serum lipid and the efficacy of external validation BCNACT patients in different ER states.

|  | pCR | | | clinical response | | | pathologic response | | |
| --- | --- | --- | --- | --- | --- | --- | --- | --- | --- |
|  | No | Yes | P value | Poor | Good | P value | Poor | Good | P value |
| Total (ER+) | 90 | 6 |  | 46 | 50 |  | 78 | 18 |  |
| FBG, mmol/L |  |  | 0.089 |  |  | 0.389 |  |  | 0.513 |
| low | 59 | 6 |  | 30 | 35 |  | 58 | 14 |  |
| high | 31 | 0 |  | 16 | 15 |  | 20 | 4 |  |
| Lipid profile, mmol/L | | |  |  |  |  |  |  |  |
| TG, |  |  | 0.635 |  |  | 0.345 |  |  | 0.594 |
| low | 61 | 4 |  | 22 | 27 |  | 17 | 4 |  |
| high | 29 | 2 |  | 24 | 23 |  | 61 | 14 |  |
| HDL-C |  |  | 0.634 |  |  | 0.621 |  |  | - |
| low | 71 | 5 |  | 3 | 3 |  | 78 | 18 |  |
| high | 19 | 1 |  | 43 | 47 |  | 0 | 0 |  |
| TC |  |  | 0.158 |  |  | **0.004** |  |  | 0.813 |
| low | 48 | 5 |  | 39 | 50 |  | 77 | 18 |  |
| high | 42 | 1 |  | 7 | 0 |  | 1 | 0 |  |
| LDL-C |  |  | 0.635 |  |  | **0.049** |  |  | - |
| low | 61 | 4 |  | 42 | 50 |  | 0 | 0 |  |
| high | 29 | 2 |  | 4 | 0 |  | 78 | 18 |  |
| Total (ER-) | 29 | 10 |  | 13 | 26 |  | 23 | 16 |  |
| FBG, mmol/L | | | 0.072 |  |  | 0.060 |  |  | **0.041** |
| low | 17 | 9 |  | 6 | 20 |  | 15 | 15 |  |
| high | 12 | 1 |  | 7 | 6 |  | 8 | 1 |  |
| Lipid profile, mmol/L | | |  |  |  |  |  |  |  |
| TG, |  |  | 0.250 |  |  | 0.210 |  |  | **0.033** |
| low | 21 | 9 |  | 6 | 17 |  | 1 | 5 |  |
| high | 8 | 1 |  | 7 | 9 |  | 22 | 11 |  |
| HDL-C |  |  | 0.410 |  |  | 0.284 |  |  | - |
| low | 23 | 9 |  | 0 | 3 |  | 23 | 16 |  |
| high | 6 | 1 |  | 13 | 23 |  | 0 | 0 |  |
| TC |  |  | 0.544 |  |  | 0.747 |  |  | - |
| low | 16 | 6 |  | 12 | 24 |  | 23 | 16 |  |
| high | 13 | 4 |  | 1 | 2 |  | 0 | 0 |  |
| LDL-C |  |  | 0.265 |  |  | - |  |  | - |
| low | 15 | 7 |  | 13 | 26 |  | 0 | 0 |  |
| high | 14 | 3 |  | 0 | 0 |  | 18 | 40 |  |

Abbreviations: pCR, pathologic complete response; ER, estrogen receptor; FBG, fasting blood glucose; TG, triglycerides; HDL-C, high-density lipoprotein cholesterol; TC, total cholesterol; LDL-C; low-density lipoprotein cholesterol.
